# MEEGIPS—A Modular EEG Investigation and Processing System for Visual and Automated Detection of High Frequency Oscillations

**DOI:** 10.3389/fninf.2019.00020

**Published:** 2019-04-05

**Authors:** Peter Höller, Eugen Trinka, Yvonne Höller

**Affiliations:** ^1^Department of Neurology, Christian Doppler Medical Centre and Centre for Cognitive Neuroscience, Paracelsus Medical University, Salzburg, Austria; ^2^Spinal Cord Injury and Tissue Regeneration Center, Paracelsus Medical University, Salzburg, Austria; ^3^Department of Psychology, University of Akureyri, Akureyri, Iceland

**Keywords:** EEG, HFO, high frequency oscillations, epilepsy, MEG, EEG software, automated HFO detection

## Abstract

High frequency oscillations (HFOs) are electroencephalographic correlates of brain activity detectable in a frequency range above 80 Hz. They co-occur with physiological processes such as saccades, movement execution, and memory formation, but are also related to pathological processes in patients with epilepsy. Localization of the seizure onset zone, and, more specifically, of the to-be resected area in patients with refractory epilepsy seems to be supported by the detection of HFOs. The visual identification of HFOs is very time consuming with approximately 8 h for 10 min and 20 channels. Therefore, automated detection of HFOs is highly warranted. So far, no software for visual marking or automated detection of HFOs meets the needs of everyday clinical practice and research. In the context of the currently available tools and for the purpose of related local HFO study activities we aimed at converging the advantages of clinical and experimental systems by designing and developing a comprehensive and extensible software framework for HFO analysis that, on the one hand, focuses on the requirements of clinical application and, on the other hand, facilitates the integration of experimental code and algorithms. The development project included the definition of use cases, specification of requirements, software design, implementation, and integration. The work comprised the engineering of component-specific requirements, component design, as well as component- and integration-tests. A functional and tested software package is the deliverable of this activity. The project MEEGIPS, a Modular EEG Investigation and Processing System for visual and automated detection of HFOs, introduces a highly user friendly software that includes five of the most prominent automated detection algorithms. Future evaluation of these, as well as implementation of further algorithms is facilitated by the modular software architecture.

## 1. Introduction

Pathological interictal high frequency oscillations (HFO) are activity in the electroencephalogram (EEG) exceeding 80 Hz. They were found to delineate the seizure onset zone in patients with epilepsy largely independently of their co-occurrence with epileptic spikes, and the resulting localization was reported to be more specifically and accurately than epileptic spikes (Jacobs et al., 2008; Andrade-Valenca et al., 2011). HFOs are said to point to the seizure onset zone more reliably than an underlying, potentially non-congruent lesion (Jacobs et al., 2009). This clinical potential was specifically emphasized for HFOs in higher frequency bands (fast ripples, 250–500 Hz; as compared to ripples, 80–250 Hz; Jacobs et al., 2008).

Apart from the considerable amount of time it takes even an expert neurologist to identify and categorize HFOs, the process is obviously prone to subjective perception and bias (von Ellenrieder et al., 2012). During recent years a number of studies elaborated on this topic and proposed concepts and algorithms for automated detection of HFOs (Kobayashi et al., 2009; Zelmann et al., 2009, 2010; Jacobs et al., 2010; Dümpelmann et al., 2012). Due to important factors, such as cost or inherent risk of invasive procedures, the question whether high-frequency oscillations are detectable using scalp EEG has been steadily moving into the focus of research. Detection of scalp HFOs is much more time consuming, error-prone and difficult than detection of HFOs in invasive recordings. Therefore, automated detection is highly warranted (Höller et al., 2018). A number of recent studies set out to detect fast oscillations non-invasively in the magnetoencephalogram (MEG) (e.g., Papadelis et al., 2016; Pellegrino et al., 2016; van Klink et al., 2016; von Ellenrieder et al., 2016; Migliorelli et al., 2017; Tamilia et al., 2017). MEG is associated with high costs and long-term or bed-side recordings are not possible. High-density scalp EEG remains an open and demanding field when it comes to analyzing actual patient data. MEG is associated with high-costs and long-term or bed-side recordings are not possible, but it offers a localization accuracy of few mm (Leahy et al., 1998; Papadelis et al., 2009). The assumed small size of cortical generators as well as the, relative to invasive data, poor signal-to-noise ratio are frequently stated as reasons for unsatisfactory HFO analysis results in scalp recordings. These factors affect the success of visual identification as well as the set of wide-spread analytical detection strategies.

Analyzing HFOs in these different types of brain signals—EEG and MEG—requires a level software support that is depending on the analysis approach. Even visual assessment based on raw waveform representation mandates as a minimum suitable bandpass filter mechanisms as well as time- and amplitude-wise scaling of data display.

So far, software support for HFO detection has been emerging mainly from two opposed directions. A number of commercial software systems are available for EEG review and analysis in clinical settings. These systems are either part of a complete EEG system from a particular manufacturer (such as “SystemPLUS Evolution”^1^ or “NetStation”^2^) or are marketed as manufacturer-independent third-party software solutions (e.g., “BESA Epilepsy”^3^ or “CURRY”^4^). The primary purpose of these systems is to assist neurologists in visually reviewing EEG recordings for pathological patterns. Most of them offer supportive tools, such as configurable frequency filters, spectral decomposition of the signal, time-frequency plots, or automated detection of epileptic spikes. Advanced functions may include the automated localization of signal sources on the basis of spikes (EGI “GeoSource”), dipole representation of electrical potentials on the scalp (“BESA”), or super-imposing implantation schemes and electrode positions upon patient MRI images (“CURRY”). Essentially, these commercial systems are tailored to integrate well with the clinical environment. The set of functions as well as the user interfaces are optimized to meet the requirements of the day-to-day workflow. Presentations of commercial products during the Freiburg-Workshop on High-frequency Oscillations in March 2016 confirmed that support for HFO analysis in these systems is typically limited to specific filter presets (e.g., band pass 80–500 Hz) and optimized data display settings. Comprehensive HFO analysis support seemed out of reach at that time.

On the other side, numerous experimental HFO analysis systems have been developed by different research and focus groups. Virtually all of them are based on “Matlab”^5^ and are frequently the results of individual or series of related studies. Consequently, development of these systems concentrated on the specific research questions, supporting the validation of concrete hypotheses, rather than on providing universal HFO research tools. Thus, the majority of these experimental approaches cannot be considered integrated systems but are actually loosely coupled sets of Matlab scripts, each of which implements a specific algorithm. “RippleLab” (Navarrete et al., 2016) which emerged into a framework that experiences a somewhat wider distribution within the research community should be mentioned as an exception.

Accordingly, the user interfaces of these systems are typically minimalistic and often reduced to the Matlab console. In order to execute a complex, multi-stage process upon the data, small programmes (scripts) have to be invoked in sequence or compiled together by means of some additional top-level script. Use and operation of these software packages is, thus, fundamentally different from commercial systems for clinical use. Usage often requires a certain level of expertise in the hosting run-time environment. Usability aspects are not within the scope of these experimental packages. Moreover, Matlab, as a domain-specific high-level environment, provides a considerable level of flexibility and a rich set of stable functional primitives at the same time, which is clearly an asset for rapidly prototyping and experimenting with new algorithms.

In view of the constant advances in understanding origin and function of high-frequency oscillations (Menendez de la Prida et al., 2015; Jacobs et al., 2016a,b; Bruder et al., 2017; Pail et al., 2017; von Ellenrieder et al., 2017) and an increasing confidence in their clinical value (Frauscher et al., 2017). It is foreseeable that all major manufacturers will integrate HFO detection and classification functionality within their commercial EEG software systems. Nevertheless, Matlab-based experimental systems will continue to play the main role in the research context, as commercial systems are usually closed and are not meant to be extended by own, experimental code and algorithms. At most they offer a limited application programming interface (API), allowing external code to interact with certain parts of the system, or data import/export functions.

In the context of the currently available tools and for the purpose of related local HFO study activities we aimed at proceeding along the path of convergence of clinical and experimental systems. We designed and developed a comprehensive and extensible software framework for HFO analysis. This framework focuses on the requirements of clinical application and facilitates the integration of experimental code and algorithms. One specific requirement was that the software is not limited to the analysis of invasive EEG, but could be used for scalp recordings, as well. Thus, the software framework should allow to analyse: (i) invasive EEG; (ii) standard low-density scalp-EEG; and (iii) high-density scalp-EEG recordings. Furthermore, a general flexibility to integrate magnetoencephalographic (MEG) data should be given.

The development project included the definition of use cases, specification of requirements, software design, implementation, and integration. The work comprised the engineering of component-specific requirements, component design, as well as component- and integration-tests. A functional and tested software package is the deliverable of this activity.

To this end, we reviewed the current literature on HFO detection in general and implemented published algorithms as modules that can be plugged into the software framework.

In the following sections we will delineate the software project MEEGIPS, Modular EEG Investigation and Processing System for visual and automated detection of HFOs. The project resulted in a highly user friendly modular software framework that is suited for both, visual and automated detection of HFOs. To date, it integrates five of the most prominent automated detection algorithms, and it can be easily extended to include newly developed algorithms. Comparing algorithms to each other was done previously (Salami et al., 2012; Zelmann et al., 2012) and is beyond the scope of this manuscript. Here, we rather focus on the framework to which algorithms can be added. The software package can be obtained via email request to meegips@pmu.ac.at. It is provided as binary application package for Mac OS under GNU Lesser General Public License v.3 (“LGPL”).

## 2. Principal Use Cases

Elaborating on the primary aim of the software framework a number of high-level use cases have been identified. In software engineering terms, a “use case” represents a defined scenario of interaction between an “actor” and the system in order to achieve a particular goal. Figure S1 depicts the interaction scenarios of our software framework by means of Unified Modeling Language (UML, Object Management Group Inc., 2015) and indicates that two “actors,” a “Neuroscientist” and a “clinical EEG specialist,” are using the system in disjoint sets of use cases. The neuroscientist and the clinical EEG specialist play specific roles. In general, “Actors” in UML define the roles that are taken on by persons or external systems when interacting with the system. They do not refer to particular user, groups of users, or professions.

While the primary intention of the EEG specialist is to compile a comprehensive report about HFO occurrence and characteristics within an EEG recording, the scientist is interested in injecting, analyzing, and optimizing HFO detection and classification strategies. Compiling an HFO report requires the EEG recording to be analyzed for HFO events, either visually or by means of automated detection mechanisms. Either approach, in turn, requires to import the recording into the system. Likewise, generating machine-learning based detection models implies the definition of suitable feature sets and the systematic analysis of detection performance during that process. Figure S1 indicates serialization of use cases as stereotyped (“include”) dependencies. As all subordinate steps can also be executed independently they are rendered as separate use cases.

Although not formalized, the use case diagram indicates that the two actors, EEG specialist and neuroscientist, are not only interacting with the system but are using the system to interact with each other. The use cases of the neuroscientist aim at establishing the most suitable system configuration that allows the EEG specialist to generate an accurate and detailed HFO report.

Summarizing the application scenarios reflected in Figure S1 we identify two actors, EEG specialist and neuroscientist, and three principal use cases:

- **UC.1** “Generate HFO report.” The EEG specialist imports an EEG recording and analyses it for HFO events, either visually or by means of analytical or machine-learning based algorithms.
- **UC.2** “Define analytical HFO detection process.” The neuroscientist defines or re-implements, integrates, and possibly optimizes an analytical HFO detection and classification algorithm. The tuned and configured algorithm is made available to the EEG specialist.
- **UC.3** “Generate machine-learning model.” The neuroscientist selects a machine-learning technique and defines an appropriate feature set. Similar to UC.2, the resulting HFO classification model is made available to the EEG specialist.

## 3. Design Objectives

While the scope of this work does not permit to reproduce the full requirements engineering process, we elaborate on the set of fundamental properties of the software framework which have been defined in accordance with the use cases determined in section 2. These design objectives serve as guidelines throughout the system design and development process.

- **Adaptability** is one of the key drivers for the in-house development. It mandates a modular architectural design, ensuring the extensibility of the system by integrating functional modules which implement particular algorithms or individual steps of staged detection procedures without the need to modify the core system. The system shall support the aggregation of functional modules into complex HFO detection and classification processes and shall allow their parameterization via store- and recallable configurations sets. For the neuroscientist this is the foundation for experimenting with and optimizing detection approaches.
- **Interoperability**. Most commercial EEG systems store EEG data in their own, proprietary file formats. In order to facilitate migration of data between systems we require that our framework is able to import EEG data produced by the locally used clinical EEG recording systems (i.e., invasive EEG, scalp EEG, low- or high-density recordings.
- **Usability** is considered the further important design objective. It refers to appropriateness of graphical user interface (GUI) as well as to clarity and comprehensibleness of activities required to trigger a particular process or achieve a certain goal. Both aspects require a balance of specificity—functionality and user interface should be tailored to the core purpose of the system, HFO detection and classification—and intuitiveness and cross-system consistency of user interfaces. The system shall not require a radical reorientation with regard to usage patterns in comparison to systems in day-to-day clinical use. As little system-specific expert knowledge as possible shall be required to efficiently use the software framework.
  In order to achieve this goal, it is essential to closely interact with the prospective user community, physicians as well as scientific personnel, throughout the development process. Incremental process models (details in section 4), early prototypes, and user involvement generating qualified feedback have proven indispensable for a system that is perceived as appropriate and useful (Eckkrammer et al., 2010).
- **Efficiency** in terms of both, execution time and development effort, is determined as a further objective. Execution time is a critical issue as high-frequency oscillations in scalp EEG reportedly occur at a very low rate (Andrade-Valenca et al., 2011), requiring extended recording times. In addition, high-density EEG supports up to 256 channels recorded with a sampling rate of at least 1 kHz. Predictive models produced by machine-learning techniques typically benefit from a reasonably large training set. The accordingly large number of patients contributes to the huge volumes of data to be processed as an additional factor.
  There are several measures to more or less accurately assess the complexity of a software system and the effort required to develop it (Sneed, 2010). Obviously, the considerable number of data processing functions and the graphical user interface are the main contributors in this case. Third party software libraries provide mathematical or signal processing related functions which are well tested and established throughout the scientific community. In order to constrain the development effort without sacrificing relevant functionality these libraries shall be integrated.
- **Platform independency**. As a last and subordinate requirement the software framework should be platform independent and should not require to be executed on any specific operating system.

In combination with the set of use cases these design objectives are the basis for deriving the user and system requirements.

## 4. Incremental Development Process

Use case-driven high-level structuring has proven beneficial particularly for planning and implementing larger scale and more complex software development projects (Tiemeyer, 2010, own experience). A phased approach supports the definition of intermediate milestones, early releases of mature precursor products with limited functional scope, and facilitates the familiarization and integration process with the operational environment.

The considered use cases of the HFO detection software framework suggest a partitioning of development activities into three successive phases, each extending the software framework by a set of related functions. Figure S1 illustrates the association of principal use cases and development phases:

- Compiling a comprehensive report on the analysis of an EEG recording with respect to HFO occurrences (UC.1) is the key use case from the perspective of the EEG specialist and, therefore, has to be supported as of **phase 1** of the development. The means of generating the essential information for the report are based on the analysis and classification of visually identified data fragments by human perception and interpretation in this phase. Automation support is limited to transforming the data of the selected fragment to time-frequency representation or spectral decomposition.
  Nevertheless, the outcome of this phase is essential for subsequent phases. It already implements functions that are fundamental also for advanced processing techniques introduced in later phases, such as importing EEG recordings created by EEG systems in clinical use, or extracting/filtering specific frequency bands from the data. In addition, the system resulting from phase 1 is needed to establish the “ground truth,” i.e. a sufficiently large, representative, and expert-validated set of HFO events within a diversified set of recordings, which serve as a reference and basis for validation of automated detection strategies.
- **Phase 2** provides initial coverage of use cases from the neuroscientist's perspective. The software system resulting from this phase allows the neuroscientist to implement and test analytical, automatic HFO detection and classification strategies (UC.2). These additional capabilities are a precondition for the subordinate use case that focuses on analysis and comparison of detection performance of different detection algorithms. Moreover, phase 2 extends UC.1, report generation, by making the tested and optimized algorithms available to the EEG specialist for determining HFO report data.
- The final **phase 3** introduces automatic HFO detection and classification based on machine learning techniques. It focuses on generating validated machine learning models for the various types of EEG data, invasively recorded, classical scalp EEG, and scalp-HD-EEG, including the definition and optimization of suitable feature sets. The required infrastructure for assessing the detection performance is already put in place during phase 2. In terms of use cases, phase 3 covers use case UC.3, machine learning based detection strategies from the neuroscientist's point of view. Furthermore, it extends UC.1, allowing the EEG specialist to utilize machine learning models for generating HFO reports.

The development phases are basically executed sequentially. Marginal overlaps, however, allow the inception of the subsequent phase in parallel to deployment of and user training for the previous one.

### 4.1. Development Process Model

Within each single phase of development, which extends from requirements specification to deployment of the intermediate product, an incremental development process model is applied. In contrast to pure sequential models that follow a strictly forward-oriented flow of activities, incremental approaches cyclically iterate the sequence of development steps. The overall functional scope of the system is divided into smaller subsets of functions each of which is specified in detail, implemented, verified and validated, and integrated with the output of previous turns, within a single iteration. The rationale of these types of process models allows to start with the design and implementation already at an early stage of the project. Even though some portions of the expected functionality may be unclear or vaguely defined, a first iteration can focus on those aspects that are agreed upon and which are fully understood by all stake-holders. In the meantime, requirements and expectations for other functions can evolve and mature in order to be processed in a subsequent iteration. Apart from being robust with regard to late or changing requirements, incremental models allow to flexibly include or exclude system functions throughout the proceeding development.

Figure S2 details the steps of the incremental process: on the basis of the applicable use cases, the subset of functions that shall be implemented in the particular iteration (the “increment”) is defined in the first step. Obviously, in the first iterations these are the functions which are well understood a priori or that are, due to technical risks, critical for the success of the entire project. The subsequent step, specification, leads to the set of requirements, which serve as the basis for the following design, implementation, and validation steps.

Each iteration results in an incomplete but functional prototype, which is made available also to the prospective users of the system for testing and evaluation purposes. These early and successively refined and extended prototypes allow a strong involvement of the targeted user community. Therefore, they allow a timely discovery of misunderstandings of use cases or requirements. The outcome of the evaluation and the resulting feedback is considered in the subsequent iterations.

After all applicable use cases of the current development phase have been covered to the agreed extent the iterative process ends with the deployment of an intermediate release of the system with a limited set of functions, integrable with the operational environment.

## 5. Top-Level Architecture

The system architecture denotes the basic structuring of the system in terms of its subsystems, interfaces between subsystems, as well as data structures that are exchanges using these interfaces. A system's architecture can be described on different levels of detail and may comprise hardware as well as software building blocks. In the scope of this project only software components are relevant. Architectural decisions are influenced by a number of parameters, such as properties of the underlying hardware platform, the operational environment, and, most important, the fundamental requirements it has to comply with. Among the requirements for this HFO detection framework, outlined in section 3, adaptability, interoperability, and usability are the key drivers for the architectural design.

**Figure 1:**
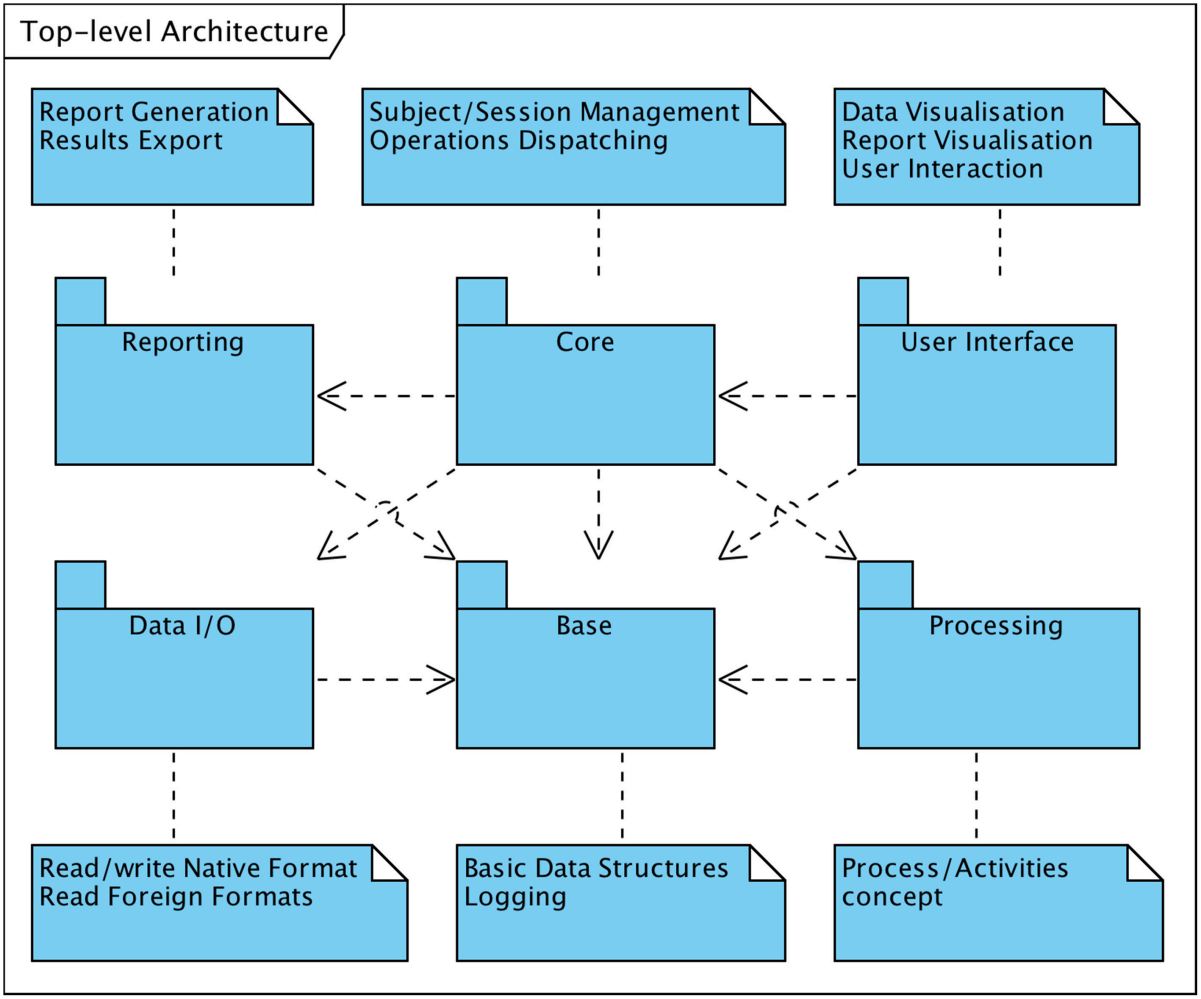
Top-level architecture in UML notation.

Figure 1 depicts the six primary constituents of the software system from the architectural perspective. All subsystems rely on the “**Base**” facility which provides fundamental data structures representing concepts such as “sessions” or “data nodes” (section 6.1). Like the “**Core**” subsystem which is the second central unit of the framework, it is a fixed part of the main component. The “Core” subsystem is responsible for subject and session management and coordinates the interaction among all further functional modules. As such it e.g., invokes and controls data processing operations whenever requested by an operator via the user interface subsystem.

All other subsystem are built upon modules that are loaded into the software system at runtime during the system's initialization phase as required. Each module implements a particular type of function in a module-specific way (polymorphism) and pertains to one of the following subsystems:

- In compliance with the requirement of interoperability with external EEG recording systems the software framework must be capable of handling proprietary data formats. The “**Data I/O**” subsystem consists of modules to import raw EEG data or MRI image sets produced by external systems.
- The “**Processing**” subsystem provides EEG data processing and manipulation functions. Modules of this subsystem implement data filtering or segmentation functions as well as all HFO-related processing, such as event-of-interest detection or event classification according to the various supported approaches.
- Usability is a crucial design criterion of the system and is primarily depending on the means for an operator to interact with the system. The “**User Interface**” subsystem enables to comprehensively control the system via a GUI. While the foundations for the user interface are built into the main component of the system, specific views onto or representation modes of the data are implemented in loadable modules.
- Functions to summarize and format information resulting from data processing activities are provided by the “**Reporting**” subsystem. The current system specification supports a single type of report termed “HFO Report.”

The outlined subsystems are not one-to-one representing separate software components, i.e., loadable modules or executable programmes. Although it is reasonable for the architectural approach to be reflected in the components structuring, multiple components may constitute a particular subsystem, such as in the systems's activities and processes concept (section 7.2). Similarly, several subsystem may reside within the same software component, such as “Base” and “Core” subsystems, both hosted in the main executable.

## 6. Interoperability

Interoperability with EEG recording systems in local clinical use is a critical aspect in order to facilitate the integration of the software framework with the every-day work-flow. The capability to import externally generated EEG recordings without prior format conversion is therefore an essential requirement. Moreover, direct access to clinical data considerably increases the amount of data instantaneously available for the purpose of testing and validating software functions.

Likewise, the software framework supports the importing of structural patient MRI (magnetic resonance imaging) images stored in NIfTI format (Neuroimaging Informatics Technology Initiative^6^), which can be linked to EEG recordings in order to define and visualize anatomic electrode coordinates.

### 6.1. Polymorphism and Inheritance

**Figure 2:**
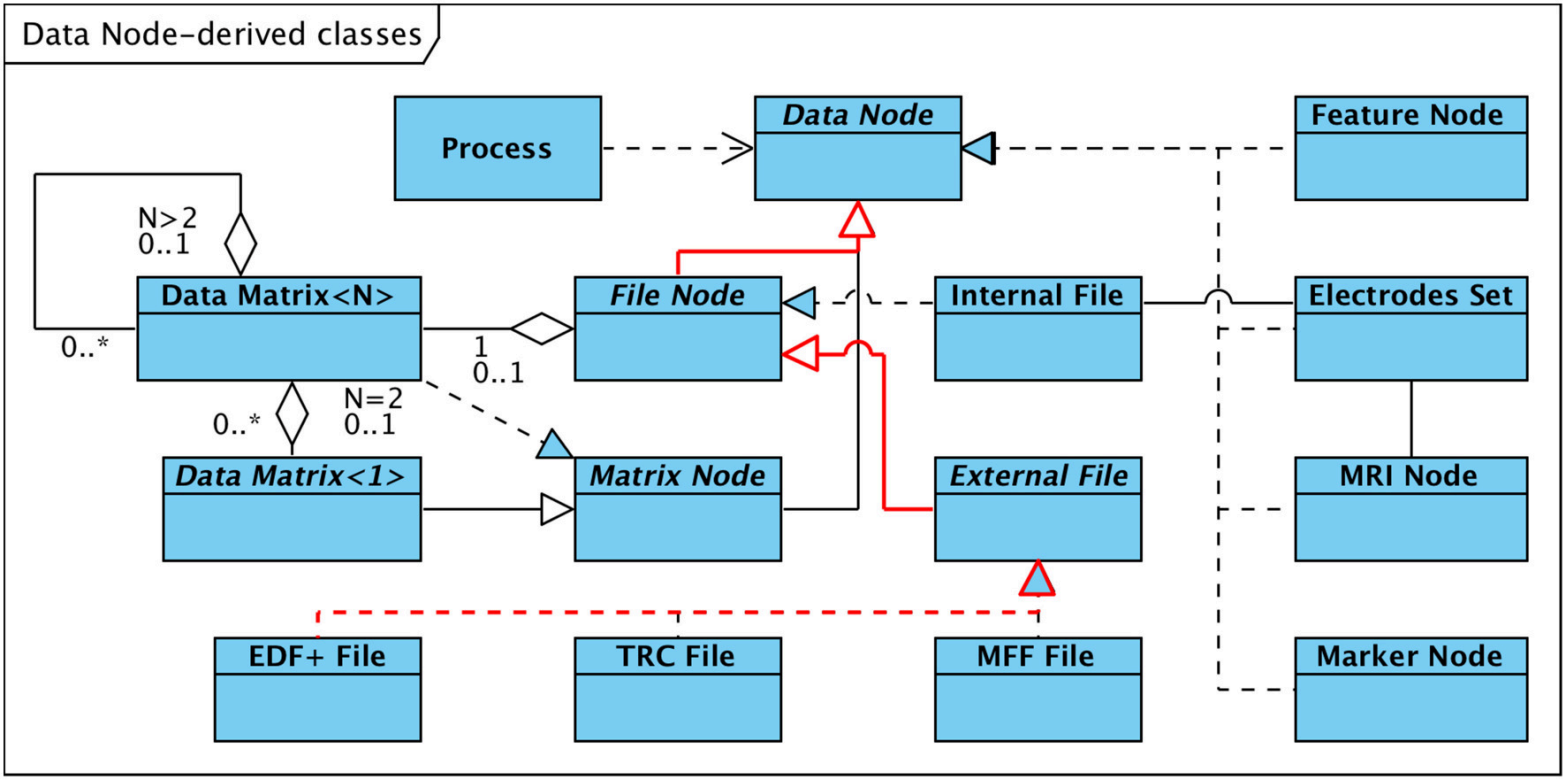
Data Structures derived from abstract Class “Data Node.” Arrows between classes denote “generalization” in the arrow direction and “specialization” in the opposite direction. The inheritance path for the “EDF+ File” class is highlighted in red.

The provision of a unified interface to data structures representing different types of data is referred to as “subtype polymorphism” and is a key concept in object-oriented software engineering (Cardelli and Wegner, 1985; Gamma et al., 1995). Our HFO software framework incorporates this technique e.g., to facilitate the implementation of interoperability in terms of reading and interpreting externally generated data. Figure 2 depicts the hierarchical organization of interfaces with the abstract class “Data Node” as the root. “Data Node” represents the top-level and most generic interface to access any kind of supported data structure, such as EEG recordings or MRI data sets. It defines the methods (i.e., operations) that other parts of the software, such as processing or visualization components, have to use in order to access data, without implementing any data-type specific mechanisms (thus the term “abstract”).

Subclasses of the abstract root class either specialize the interface by defining additional methods more trimmed to the type of data they represent, or implement (or “realize”) the interface, i.e. provide the actual data manipulation mechanisms and data-type specific code.

Figure 2 details the hierarchy on the example of externally generated EEG recordings in EDF+ (European Data Format^7^) format; the relevant dependencies are highlighted in red: A set of specialized subclasses of “Data Node” represent the first specialization step. The subclass “File Node” extends the generic “Data Node” interface by methods that are more specialized to access EEG data that is persistently stored in a file. “File Node” allows users of this interface to access/read EEG data without the need to know the details of how signal and meta data are structured and arranged within the file. The next level of specialization distinguishes between “Internal File,” a “realization” implementing read and write access to EEG data stored by the software framework in its internal data format, and “External File”. “External File,” in turn, extends the “File Node” interface by methods to read meta information that is not part of internal data files, such as subject, epoch, trigger, or event data associated with the recording. It defines the most specific interface for accessing EEG data stored in external data formats. Currently the following three realizations of the “External File” interface are available (see also section 6.1):

- The **EDF+ File** class discussed in the example above implements read-only access to EEG data stored in either type (binary and textual) of EDF+ file.
- Long-term EEG recording in the local epilepsy monitoring unit (EMU) is based on a system manufactured by Micromed, Italy. The system stores EEG data and associated meta information in files according to a proprietary format (TRC, “TRaCe file”). Support for read-only access to TRC (version 4) formatted data is provided via the **TRC File** class.
- Read-only access to MFF (“Meta File Format”) formatted EEG data generated by EGI (Electrical Geodesics Inc.) high-density EEG systems is implemented in the **MFF File** realization of the “External File” interface.

The obvious advantage of the hierarchical data abstraction is that software entities can access EEG data in a generic way without needing to know the actual structure and format of the file the data is stored in. This way it is possible to add support for additional external data formats without impacting portions of the software that access the data using the generic interface.

### 6.2. Matrix-Based Representation of Data

A critical aspect with respect to interoperability is the encapsulation of EEG data within constructs that are independent of the format in which the data are physically stored on the persistent medium (typically hard disk), while at the same time accurately reflecting the logical structure of the data, i.e., its partitioning into channels and epochs. Moreover, in order to ensure efficient processing of the data, a direct and random access to individual data elements (samples or measurement values of a certain channel at a particular point in time) has to be facilitated.

For that purpose the internal data storage concept of the software framework is based on virtual matrices of arbitrary dimension. Successive samples of a single channel are stored as a matrix of dimension (or order) one, that is, a contiguous vector or array of data values. In case of multiple simultaneously recorded channels the dimension of the matrix is increased to accommodate the set of channels. The typical representation of a recording covering a single epoch therefore results in a matrix of dimension two. A crucial precondition for the organization of individual channels within a single matrix is that their samples cover the same span of time. Whereas, the sampling rate and, thus, the number of samples per channel may differ.

Recordings that consist of multiple epochs require the extension of the matrix to an order of three, with the third dimension covering the epochs. The order of the data matrices can be set as required. For example a set of recordings of the same subject, each consisting of multiple epochs and multiple channels would be organized as a single four-dimensional matrix.

**Figure 3:**
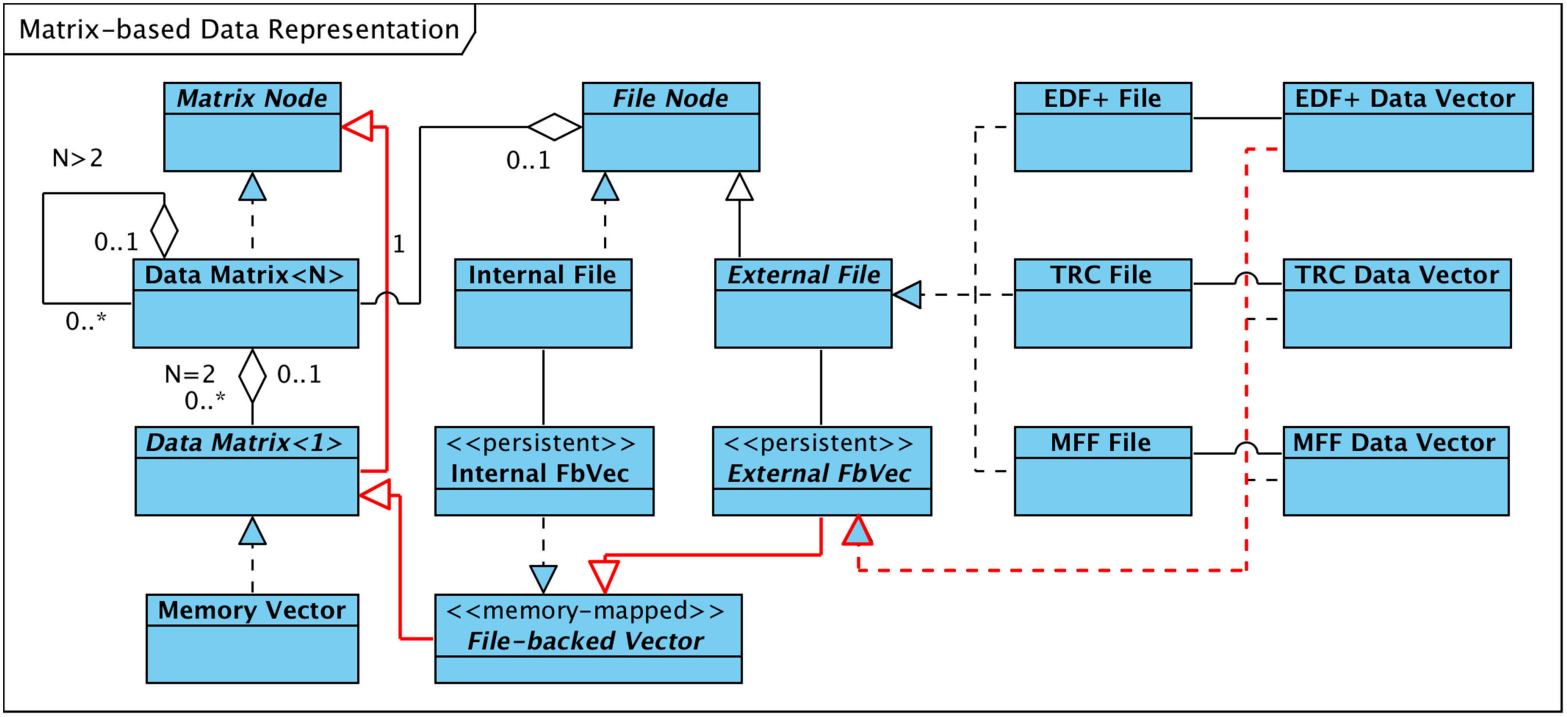
Descendants of abstract Classes “Matrix Node” and “File Node.” Arrows between classes denote “generalization” and “specialization” relationships. Inheritance Tree for EDF+ Data Vector Class in red.

Figure 3 visualizes the concept. An instance of the template class “Data Matrix” of dimension *N* holds a vector of instances of the template class of dimension *N*−1 and may or may not have an association with an object of type “File Node” (indicated in the figure as a containment or “part-of” relationship of multiplicity 0 or 1). Matrices of dimension one (i.e., vectors) require special consideration since they are responsible for adapting the format-dependent physical data storage to the uniform vector-based structuring. Accordingly, these vectors are implemented by a set of classes. They either represent an array of measurement data that is available in the working memory of the computer, denoted “Memory Vector” in Figure 3, or enable access to data stored in internally or externally generated persistent data files (“File-backed Vector”).

As outlined in Figure 3 a specific implementation of the “File-backed Vector” interface manages read/write access to data stored in internally generated EEG data files (“Internal FbVec”). For data files in proprietary third-party formats, a more specialized interface is derived from “File-backed Vector,” labeled “External FbVec”. This interface is realized by three classes, each of which transforms the vendor-specific layout of the EEG data of the individual channels stored within EDF+ files, TRC files, or MFF files, respectively, into a linear, contiguous data vector. Figure 3 highlights in red the exemplary inheritance hierarchy for the “EDF+ Data Vector” case.

In both cases, “Internal FbVec” implementation or realizations of “External FbVec,” the data that are physically kept in files on the hard disk are mapped into the logical address space of the process executing the HFO software framework using the computer's Memory Management Unit (MMU). This technique, called “memory-mapping,” allows a process to access the data as if it were available in the computer's main memory, regardless whether the data are actually in memory or on disk. As a major benefit memory-mapping enables direct access to data values at arbitrary positions without prior allocation of buffer storage that needs to be filled with the proper portion of data loaded from the file. Buffer management and optimization is entirely left to the operating system.

## 7. Adaptability

An aspect substantially driving the in-house development of a software framework for HFO detection is the high level of flexibility which is required for experimenting with, testing, and validating novel HFO detection and classification strategies. Tools that enable the definition, revision, and parameterization of detection processes are an important requirement and precondition for an adaptable framework. A comprehensive concept of configurable processes aids in achieving this flexibility.

### 7.1. Process Structure and Data Flow

**Figure 4:**
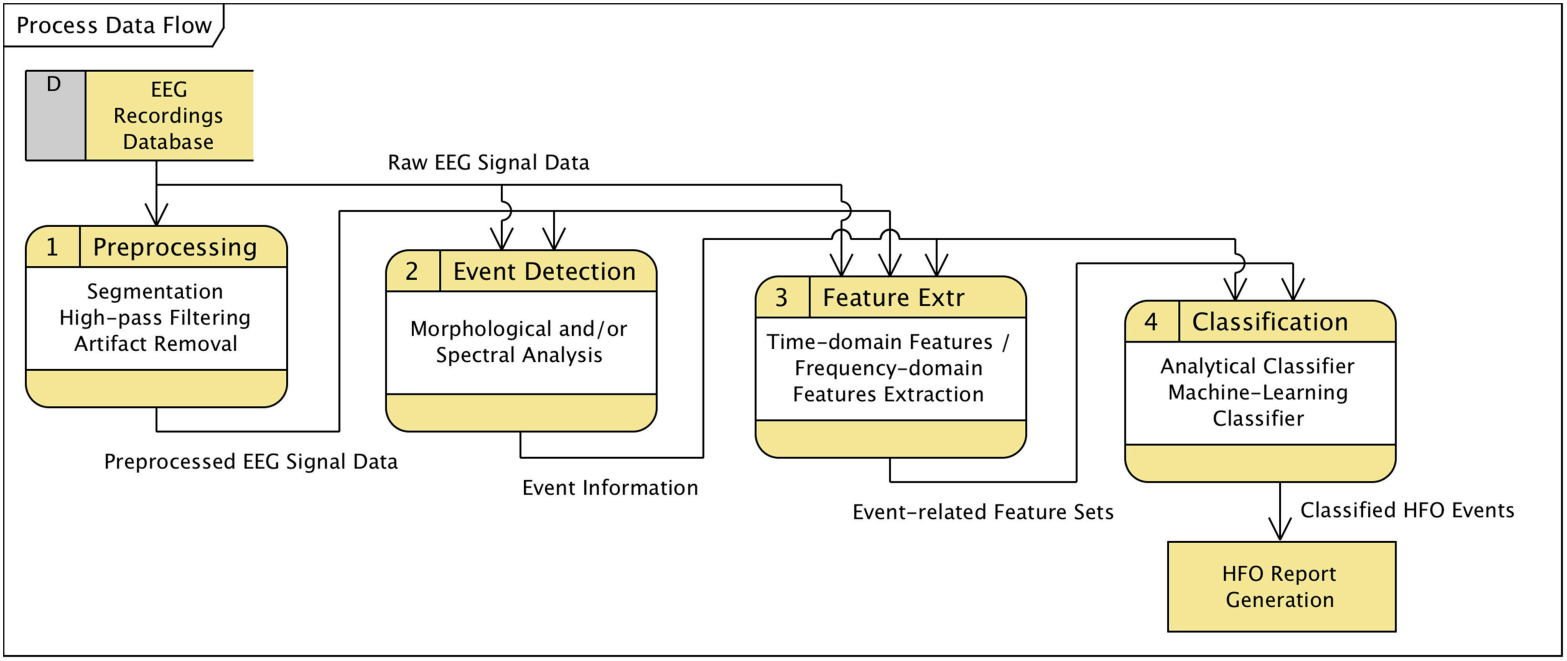
Data flow diagramme (DFD) covering steps of an HFO detection and classification process.

In general, HFO detection and classification strategies, regardless whether following analytical approaches or utilizing machine-learning techniques, can be broken up into sets of sequentially executed processing steps (see also Zijlmans et al., 2017). The data flow diagramme in Figure 4 illustrates the typical functional decomposition that is followed by our software framework and indicates the data items that are passed between consecutive processing steps.

In the **preprocessing** stage the original EEG recording is band-pass or high-pass filtered and potentially subject to artifact detection and removal. If only particular portions of the EEG data shall be further processed, preprocessing may include a segmentation of data according to defined types of associated events or specified time-ranges. The preprocessed data is passed on the next step, “event of interest” (**EOI**) **detection**. Depending on the algorithm, EOI detection may additionally rely on the original (unfiltered) EEG signal, which is provided as auxiliary input data. Detection algorithms that are well-balanced in terms of sensitivity and specificity may already yield an acceptable fast oscillation detection performance. However, the purpose of the EOI detection steps enclosed in the process definitions of our software framework is to preselect potential HFO occurrences by looking at morphological (time-domain) and spectral (frequency-domain) properties of the preprocessed and optionally raw signal data. The scope of subsequent processing steps is reduced from the entire recording (or selected segments thereof) to a set of time-bounded fragments of data. That is, it is reduced from continuous data to discrete sections of short duration, significantly decreasing the computational effort required by feature extraction and classification operations.

The set of EOIs serves as a database of time/channel records for the following steps. The **feature extraction** stage separately analyses the referenced sections of data with respect to time-domain or frequency-domain characteristics (features), using preprocessed and/or the original signal data. The resulting output, a map that associates each event of interest with the extracted properties at the specified section of data, is forwarded to the **classification** step. Classification evaluates the features either analytically or utilizing machine-learning techniques and classifies the events accordingly into fast oscillations or artifacts.

Although this theoretical concept considers detection and classification as separate stages, process definition and parameterization requires a sound understanding of the inter-dependency of the implemented algorithms. The upper bound for the sensitivity of the entire process, for instance, is obviously predetermined by the detection step; the classifier has no means to compensate for a high type II error (false negatives) introduced by the detecting stage.

### 7.2. Configurable Processes

A process in terms of our software framework is composed of a sequence of individual processing steps (“activities”) that are executed upon the data in a well-defined order and which incorporate each a specific algorithm or part thereof. Each processing step is implemented as a loadable and exchangeable software module and can be initialized with either pre-defined or operator-defined parameters. In this manner complex processes can be defined and parameterized based on an extensible set of simple and combinable steps.

**Figure 5:**
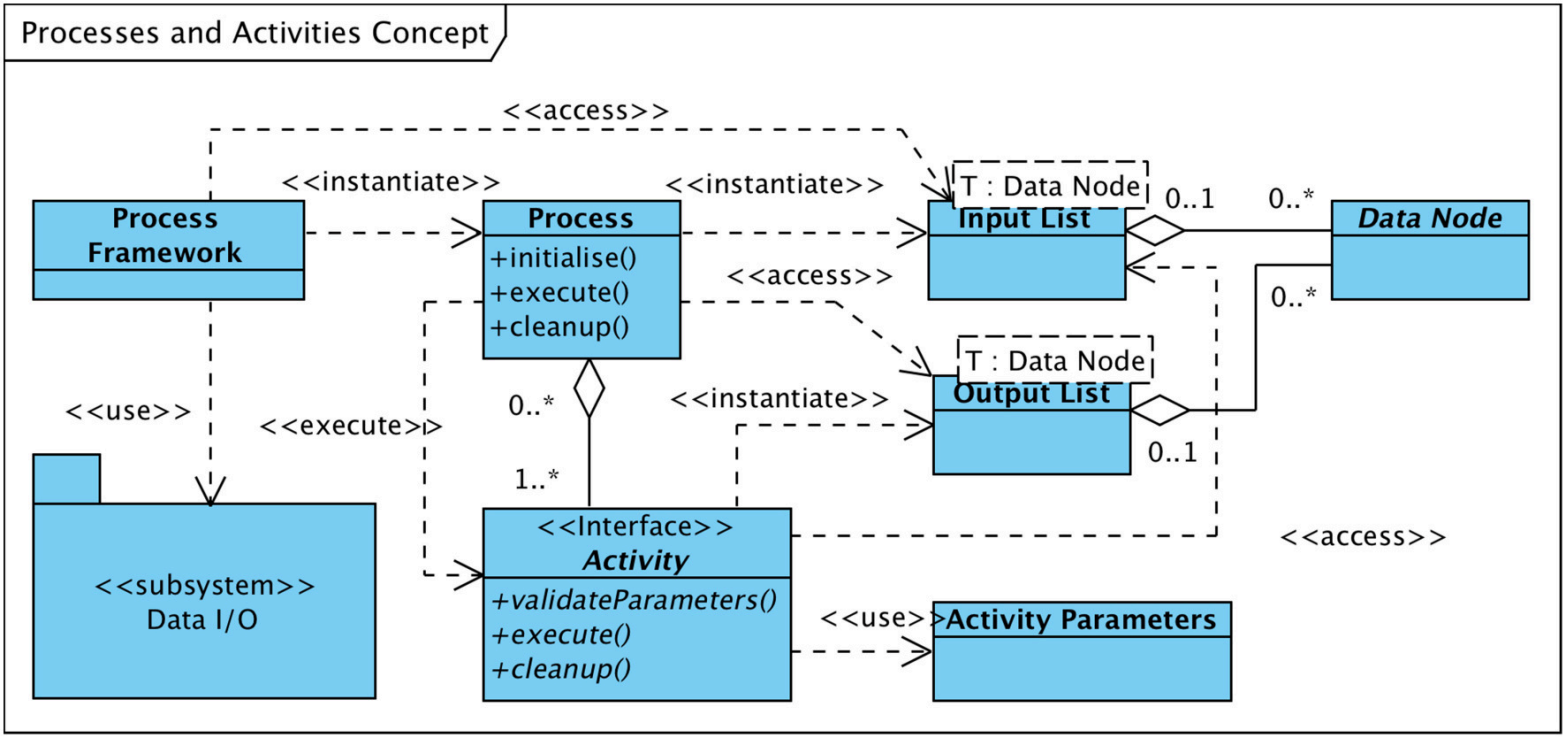
UML class diagramme of processes and activities concept.

Figure 5 visualizes the concept. Each process is subject to a process-lifecycle that is controlled by the process framework and which is in charge of instantiating, parameterizing, invoking, and removing processes. A process comprises at least a single “Activity.” All activities of a process are executed in a defined sequence and are provided with the applicable set of activity-specific parameters, which has been previously loaded or defined by the operator.

The primary data set that shall be processed by an activity as well as possible auxiliary data elements required in support of the operation to be performed are provided to the activity by means of a list of input data items. These data items may be of any type that is derived from the “Data Node” class (see also Figure 2), i.e., raw or preprocessed EEG data, sets of events or markers, or extracted features. The initial list of input data nodes is created by the process framework upon process initialization and contains data nodes which either reside in working memory or are stored on the hard disk. In the latter case, the “Data I/O” subsystem, one of the major architectural building blocks (ref. section 5), is utilized to access the data.

New data that may be generated by the executed activity is as well encapsulated in data structures subclassed from “Data Node” and are returned to the invoking process as a list of output data nodes. The process appends the list of output data nodes received from the activity to the list of input data nodes passed on to subsequent activities, enabling activities to use data structures created by preceding activities. The general approach is to not modify input data nodes in the course of activity execution but to create new (output) data nodes holding the modified input data.

After the process has completed (i.e., all activities finished successfully) the process framework adds all or a subset of the newly generated data nodes to the list of sub-nodes of the primary input data node—as per process definition. By means of the Data I/O subsystem these new nodes are copied to persistent data storage.

#### 7.2.1. Process Definition

**Figure 6:**
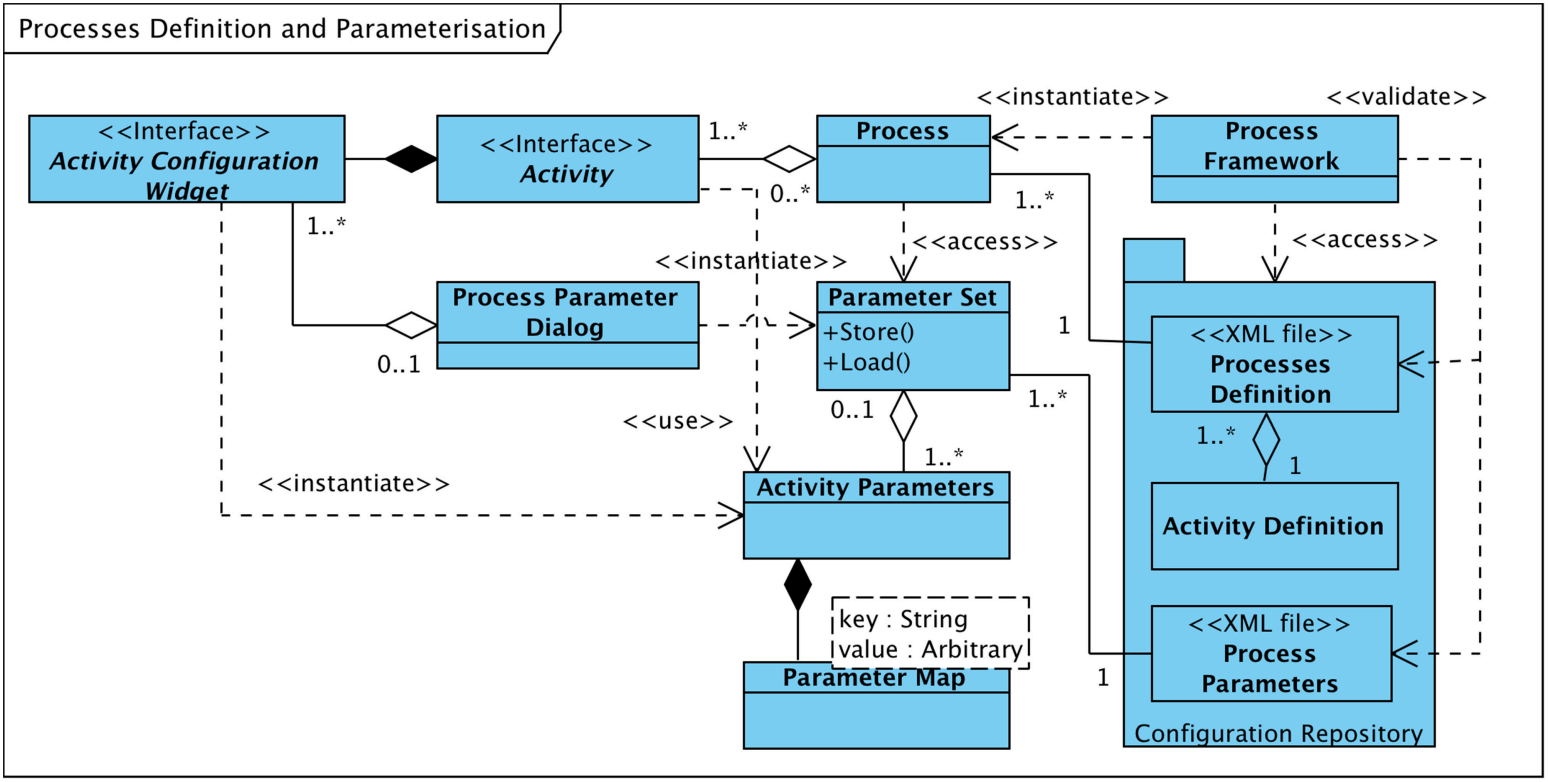
Class diagramme: process definition and process parameters overview.

Processes are aggregations of activities that are executed strictly in sequence. The set of activities and their order is statically defined in the “process definition file.” At system startup the process framework parses the process definition file and instantiates the processes in accordance with their “building plans.” Apart from a process' structure this file also defines the set of input data nodes each of its activities receives upon execution. Figure 6 gives an overview of processes, activities, and the defining structures residing in the system's “configuration repository.”

The process definition file is based on XML^8^ and internally structured as detailed in Figure S3. The first section of the file (“moduleList”) holds the list of activity-modules that shall be dynamically loaded during the software framework's initialization phase. Each module is described by a textual identifier (“moduleName”) via which it can be referenced from within activity definitions and the module's “path” (i.e., its storage location on the hard disk). The subsequent section contains the definitions of the individual processes. Apart from an element that can be used to refer to literature the process is based upon (“reference”), each process definition holds the sequence of associated activities, identified by their names, as well as a (possibly empty) list of “Data Node XML” items (“outputList”) that shall be stored as the process' final output. Each of these data node items refers to a specific type of result of a particular activity. In accordance with data node inheritance tree outlined in section 6.1 data nodes can be classified as either signal data nodes, markers or events nodes, or feature nodes.

Activities are defined by “Activity XML” elements. Such element contains the activity's textual identifier (“activityName”), a reference to the software module that implements the activity, and a list of “Data Node XML” items (“inputList”) that specify the data nodes the activity receives as input.

#### 7.2.2. Process Parameterization

Most activities of a process can be configured through an arbitrarily large set of parameters that can be adjusted within the respective ranges of values. Adjustment of parameters is conducted by the user by means of the software framework's graphical user interface. For this purpose, each activity module provides a custom dialogue page (“Activity Configuration Widget,” see Figure 6) which lists the parameters of the implemented processing step and their current values. The dialogue pages of all activities of a process are aggregated by the “user interface” subsystem (see section 5) into an interactive dialogue (“Process Parameter Dialog”) in order to provide a comprehensive overview of the parameter set and to allow the user to modify the parameters' values within their respective boundaries.

So as to have a reasonable baseline for the parameterization of processes, default parameter sets that are typically derived from the values used in the literature are prepared for all defined processes and are pre-stored in the Configuration Repository of the Software Framework. In addition, a complete set of modified parameters can be persistently stored and recalled for subsequent use. After parameter adjustment or recall is completed a “Parameter Set” object is generated from the current values, which is made available to the process and its activities during execution.

### 7.3. Processes and Activities

**Figure 7:**
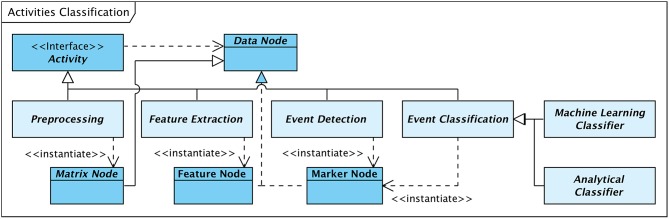
Logical classification of activities.

Although modules implementing activities follow a unified technical structure and are not differentiated by design, activities can be classified according to their purpose, type of operation, or stage within an embracing process. The type of output or result that is generated by an activity can be considered as an additional discriminating criterion. Figure 7 gives an overview of a logical classification of activities with particular focus on HFO detection.

In accordance with Figure 4 activities can be classified as follows:

- The class of **preprocessing** activities comprises all operations that prepare or preprocess the input signal data in a way required or suitable for the subsequent processing steps. This class of activities includes e.g., filtering or data segmentation operations. The generated output is typically a modified version of the input signal data, thus, again a “Matrix Node.”
- Activities that analyse and scan the input data for events of interest can be classified as **event detection** activities, with a context-specific definition of EOI. In the scope of HFO detection, EOIs are defined as signal data fragments that possibly contain high-frequency oscillations. The result of a detection activity is a data node that contains a list of event markers (“Marker Node”), each identifying a detected event of interest.
- **Feature extraction** denotes the class of activities that analyse those fragments of the input signal data that have previously been marked as events of interest. Feature extraction operations explore the signal with respect to specific properties, either based e.g., on a morphological analysis, a spectral decomposition, or a time-frequency transform of the signal. The analysis results in an arbitrarily large set of properties or features per EOI. These feature sets are encapsulated in an output element of type “Feature Node.”
- **Event classification** operations review events of interest detected by an event detection activity and classify the events according to a defined scheme. In the framework of HFO detection, classification activities differentiate EOIs between ripples, fast ripples, and artifacts.
  As outlined in Figure 7 classification activities can be further grouped into **analytical** methods and **machine-learning** based approaches. Analytical algorithms analyse and classify EOIs on an individual basis. The signal characteristics considered depend on the implemented algorithms and may include time-domain as well as frequency-domain aspects. Classification is typically performed according to some theoretically founded thresholds.
  Machine-learning classifiers, in contrast, classify events based on a previously generated “prediction model” in combination with the set of properties extracted by preceding feature extraction activities.

All activity modules have to implement the “Activity” interface in order to provide to the process or process framework a generic means of controlling the operational state of an activity and to supply it with the required set of parameters.

This classification of activities is in line with the conceptual process decomposition presented in Figure 4. Nevertheless, separate processing steps are not necessarily contained in dedicated modules. As implemented in the current system, analytical classifiers combine their own feature extraction operations and the actual classification steps in integrated modules.

#### 7.3.1. Implemented Activity Modules

Table 1 details a selection of available activity modules. These modules have been implemented in course of reviewing relevant publications on automated HFO detection strategies. They either represent the building-blocks of algorithms that, according to the respective authors, produced promising results in comparison to an expert-validated ground truth, or are the results of experimental optimizations and research into complementary methods.

**Table 1:** Selection of implemented activity modules.

**Activity**	**Type**	**Technique**	**References**
FIR filter	Preproc	Finite Impulse Response (FIR) filter using Blackman-windowed Sinc-kernel.	Smith, 1998
EMD filter	Preproc	High-pass filtering based on empirical mode decomposition (EMD), signal recomposition based on a subset of lower-order intrinsic mode functions.	Flandrin et al., 2004
RMS	Detector	Root Mean Square (RMS) calculation within sliding window. RMS values > n times standard deviation (SD) above mean, considering minimum duration, and minimum number of peaks.	Staba et al., 2002
Hilbert envelope	Detector	Envelope of band-passed signal using Hilbert transform, SD of envelope > n times SD above mean, considering minimum duration, and minimum number of peaks.	Burnos et al., 2014
Stockwell transform	Classifier	Analysis of blobs of high amplitude around max. amplitude of time-domain signal within EOI in Stockwell transformed data. High-frequency peak between 60 and 500 Hz, low-frequency peak = closest local maximum below trough.	Stockwell et al., 1996; Burnos et al., 2014
Bump modeling	Classifier	Z-score normalization of complex Morlet wavelet divided into set of time-frequency windows. Parameterization of windows by half ellipsoid functions modeling the HFOs.	Vialatte et al., 2009; Doshi, 2011
SVM	Classifier	Support Vector Machine (SVM) classifier (“libsvm”) based on Gaussian radial basis function kernel. Model generation using 3-stage cross-validation, optimization of feature subset, cost and gamma.	Chang and Lin, 2011; Hsu et al., 2016
Time-domain features	Feature extraction	Extracts various properties from the time-domain representation of the signal.	Blanco et al., 2010; Pearce et al., 2013; Amiri et al., 2016
Frequency-domain features	Feature extraction	Extracts various properties from the frequency-domain transformed signal.	Blanco et al., 2010; Matsumoto et al., 2013; Pearce et al., 2013

*Preproc, preprocessing activity*.

Further implemented activity modules not listed in the table include test signal generators, event matching and reclassification operations, or signal segmentation modules.

#### 7.3.2. Predefined Processes

On the basis of the implemented activity modules, a set of HFO detection and classification processes has been defined, either in strict accordance with the reviewed publications or as recombinations of processing steps suggested in the literature. The selection of analytical detection algorithms is primarily influenced by comparative benchmarks performed in Zelmann et al. (2010); Chaibi et al. (2013), and Burnos et al. (2014).

Table 2 lists the predefined processes and their constituting activities. These processes together with default sets of parameters are ready to be applied to input EEG data in one of the supported recording formats (see section 6.1).

**Table 2:** Predefined HFO detection and classification processes.

**Process**	**Activities sequence**	**References**
FIR, Hilbert envelope, power spectral density analysis	[P] FIR filter [D] Hilbert envelope EOI detection [C] Stockwell-transform classifier	Burnos et al., 2014
FIR, RMS, power spectral density analysis	[P] FIR filter [D] Root mean square EOI detection [C] Stockwell-transform classifier	Staba et al., 2002; Burnos et al., 2014
EMD, RMS, power spectral density analysis	[P] Empirical mode decomposition [D] Root mean square EOI detection [C] Stockwell-transform classifier	Staba et al., 2002; Flandrin et al., 2004; Burnos et al., 2014
FIR, RMS, bump modeling	[P] FIR filter [D] Root mean square EOI detection [C] Bump modeling classifier	Staba et al., 2002; Vialatte et al., 2009; Doshi, 2011
FIR, RMS, SVM	[P] FIR filter [D] Root mean square EOI detection [F] Time-domain feature extraction [F] Frequency-domain feature extraction [C] Support vector machine (SVM) classifier	Staba et al., 2002; Chang and Lin, 2011
FIR, Hilbert envelope, SVM	[P] FIR filter [D] Hilbert envelope EOI detection [F] Time-domain feature extraction [F] Frequency-domain feature extraction [C] Support vector machine (SVM) classifier	Staba et al., 2002; Chang and Lin, 2011; Burnos et al., 2014

*[P]: Preprocessing stage*.

*[D]: EOI detection stage*.

*[C]: Classification stage*.

*[F]: Feature extraction stage*.

### 7.4. Behavioral Model of Processes

Behavioral models describe the dynamic aspects of a software system: its activities, communication via internal and external interfaces, or state machines of the entire system and its constituting components. UML provides a class of diagrams supporting behavioral analysis and dynamic modeling as observed from different perspectives and with focus on particular facets.

The activity diagram in Figure S4 details the flows of control and data during the execution of a defined process consisting of a sequence of activities. It can be considered as complementary to Figure 5, which elaborates on the structural aspects of a process while the Figure S4 details the generic behavior of a process being executed. The activity (“Process::execute”) commences with checking whether the “Input Data Node” received from the process framework is a valid data matrix and setting up of the “Input Matrix Vector” with that initial element. In the case of HFO detection the input data node is the recording to be analyzed with respect to HFO occurrences.

The core of process execution is the iterative invocation of its activities according to the defined sequence (“Chain of Activities”). Before an activity is actually executed, it is attached to the “Progress Monitor” which allows the software framework, and the system's user, to keep track of the status of the activity. Each activity internally follows the classical IPO (input – processing – output) model and generates output data by processing its input data in a specific way. If the activity completes successfully the produced output (“Output Data Node” in Figure S4) is appended to the input matrix vector in order to be used by subsequent activities. The loop ends either after the last iteration, i.e., execution of the last activity in the sequence completed, or as soon as an activity failed to successfully execute. In the latter case an “Error Vector” is assembled that summarizes the reasons for failure and provides respective feedback to the user. If, in contrast, the entire process completed successfully the resulting “Output Data Node” is handled according to the process definition (“outputList” element in Figure S3). It may be copied to persistent storage and is either registered as a new sub-node of the input data node in case the output is of type data matrix, or is associated to the input node as a set of event markers, or both.

Additionally, the process framework allows to store intermediate results of a process, i.e., output data nodes created by other than the last activity in the sequence, in order to make them accessible for display and review. An option that can be configured by means of the process definition.

### 7.5. Integration of Custom Modules

One of the key assets of the software framework is its support for extending (HFO detection and classification) processes by new activities and for defining entirely new processes and activity sequences. Activities in terms of the software system are implemented in dedicated software modules (dynamic libraries) that are loaded on demand, i.e., in case the implemented activity is referenced by any defined process, at system startup time.

Figure S5 illustrates the fundamental structure an activity module has to comply with in order to be integrated with the software framework. Primary constituent of the module is its main activity class, denoted “Activity A” in this exemplary outline, a concrete realization of the “Activity” interface. From the set of methods that have to be backed by activity-specific implementations, “execute” and “parameterTab” deserve a close look. The “parameterTab” method instantiates and provides a configuration object, derived from the “Activity Configuration Widget,” that lists all parameters relevant to the implemented algorithm for review and modification by the user. The methods it provides are used to convert the activity-related section of a “Parameter Set” (see Figure 6) to and from a set of Graphical User Interface (GUI) elements.

The “execute” method is invoked by the hosting process. It actually performs the implemented operation on the input data, using the previously adjusted parameters. There is no interaction between activity and process framework, and in particular no concurrent access to the processed data from outside the activity, until the “execute” method has completed and returns control to the embracing process. Thus, the activity can autonomously schedule and organize access to the data as best suited for the applied algorithm. While, for instance, frequency-band filter activities are likely to work on a per channel basis, some artifact detection techniques may require to access data across channels. This opens up the possibility to delegate processing of defined portions of the data, e.g., channels, to a pool of concurrent threads (“Channel Processing” class in Figure S5). Depending on the hosting hardware platform, this may lead to a considerable reduction of execution time.

The classes stereotyped “Qt” are base classes provided by the embedding cross-platform application framework and link the elements of the activity module into the framework (see section 9 for more information). Modules are independently constructed and compiled and represent separate binary components which can be modified without impact on other components of the HFO detection software framework. Eventually, this enables interested external institutions to extend the HFO detection framework by their own algorithms and experiments.

## 8. Integration of Machine-Learning Techniques

In most general terms machine learning can be described as the capability of a technical system (i.e., a computer programme in this context) to adapt its behavior or decision making according to a history of input data—it aims at generalizing on the basis of provided examples or, less technically, it “learns” from “experience.” Machine learning algorithms typically analyse training data sets for intrinsic patterns or rules and construct predictive models under the assumption that discovered patterns are valid for the entire population.

Machine learning techniques can be a valid alternative to direct, analytical programme design in case of ill-posed problems. Ill-posed problems defy an exhaustive and precise description by analytical algorithms and can be better explained by means of representative examples. A frequent inherent characteristic of such problems is a high level of complexity, a high number of degrees of freedom, and an insufficient understanding of their interrelationship. A further, related indication for machine learning approaches is the analysis of data for structure-defining features that are obscured by more prominent properties or the sheer volume of data (see also Nilsson, 1998; Shalev-Shwartz and Ben-David, 2014). Indeed, most of these factors apply to the problem of HFO detection in invasive and particularly scalp EEG recordings, not least reflected in the poor inter-rater agreement rates reported e.g., by Blanco et al. (2010), rendering HFO detection a candidate for the potentially successful utilization of a machine learning-based approach.

Machine learning techniques can be typed based on a number of aspects, among which the provision of the correct function values or labels associated with the training data, i.e., supervised vs. unsupervised learning, is the most distinctive one. Supervised learning aims at evaluating properties of the training data in order to support the underlying hypothesis that is represented in the association of provided training samples and provided values or labels. The learning process establishes a relation between given properties of the data and given labels. Unsupervised learning, in contrast, analyses the training data without any a-priori knowledge of the structure of the data. The unsupervised learner is provided the (number of) labels only without association to training data samples and autonomously attempts to discover intrinsic structural characteristics in order to partition the data into meaningful clusters.

A large class of applications of supervised machine learning is the automatic classification of data instances. The classification of EOIs into ripples, fast ripples, and artifacts in course of HFO detection in EEG recordings is one such application. Considering the data flow diagramme in Figure 4, classification of detected events is the last stage of the conceptual detection and classification process. Accordingly, in our software framework machine learning-based classification can be configured as the final step (“Event Classification”, see Figure 7), as a particular type of classifier, of an HFO detection and classification process.

### 8.1. Support Vector Machines

The publications on machine learning in HFO detection use four different techniques to approach the problem (see Figure S6). Studies going for a supervised method apparently favor support vector machines (SVM), while only a single publication is using k-nearest-neighbor (k-NN) and logistic regression models in addition.

Support vector machines have been preferred over k-NN as a machine learning classifier model for integration with our HFO detection software framework for mainly two reasons: As detailed in Kim et al. (2012) and Kaushik and Singh (2013) k-NN models are very sensitive to the choice of the parameter “k” and the used measure of “distance” between any two samples. In applications resulting in higher-dimensional feature spaces k-NN performance deteriorates in terms of both, classification and execution time. In addition, outliers tend to excessively influence the classification accuracy as all data values contribute to the result to the same extent. In particular, for a large number of features even in combination with a small number of samples SVM have proven to yield better classification results as long as the margin (i.e., the geometric “distance”) between classes is sufficiently large. The second reason for a decision in favor of SVM is the availability of open-source SVM libraries (“LIBSVM” and “LIBLINEAR,” see Chang and Lin, 2011) that can be easily linked into a classification module/activity.

### 8.2. Construction of Feature Vector

Besides the decision for a suitable machine learning model, the diligent construction of the feature vector is of critical importance for the success of the learning algorithm. The careful selection of those input data properties which are assumed to have the most discriminative power with regard to the intended classification considerably influences the quality of the resulting predictive model. Not even the best type of learner is able to compensate for poorly chosen features. Selecting reasonable features requires a certain understanding of the nature and characteristics of the input data. This type of a priori information, also called “bias” or “prior knowledge,” is a vital prerequisite for any useful learning process (Nilsson, 1998; Shalev-Shwartz and Ben-David, 2014).

The events of interest to be classified in our application are fragments of short duration of a digitized, that is, a quantized and discretized EEG signal. Such signal can be analyzed with regard to its morphological, time domain properties (observing the signal as a function of amplitude over time), as well as considering its spectral composition (in terms of proportional contributions of oscillations of distinct frequencies to the original signal). In addition to the review of features used in earlier studies using machine learning, the in-depth assessment of published analytical approaches is of particular advantage for the feature vector definition. Analytical methods are basically considering the same distinctive signal characteristics, only the processing strategy is entirely different.

#### 8.2.1. Time Domain Features

The HFO detection software framework implements the extraction of time domain features within a dedicated module (class “feature extraction” in Figure 7) that can be registered as one step of a process' sequence of activities. The time domain feature extraction module receives a data matrix node, i.e., the high- or band-pass filtered EEG signal, and a list of events of interest to be processed as input data. Each event is analyzed with respect to the signal characteristics detailed in Table 3.

**Table 3:** Implemented time domain signal features.

**Feature description**	**Acronym**	**References**
Average segment amplitude; mean value of all samples of segment ($\bar{a}$).	mAmp	Blanco et al., 2010
Amplitude delta between segment maximum and segment minimum; peak-to-peak over segment.	dAmp	Pearce et al., 2013; Amiri et al., 2016
Average time ($\bar{dt_{ex}}$) between consecutive local extrema.	dtEx	—
Duration of event of interest.	dur	Matsumoto et al., 2013; Amiri et al., 2016
Maximum positive gradient; steepest transition from local minimum to local maximum.	gdPos	Amiri et al., 2016
Maximum negative gradient; steepest transition from local maximum to local minimum.	gdNeg	Amiri et al., 2016
Maximum amplitude relative to mean amplitude ($a_{max}-\bar{a}$).	rmaxA	—
Minimum amplitude relative to mean amplitude ($a_{min}-\bar{a}$).	rminA	—
Total line length of segment ($\underset{T}{\sum }|a_{t}-a_{t-1}|$).	linLen	Gardner et al., 2007; Blanco et al., 2010; Pearce et al., 2013
Number of local extrema.	numEx	Pearce et al., 2013; Amiri et al., 2016
Standard deviation of local amplitude extrema relative to mean amplitude ($sd\left(a_{ex}-\bar{a}\right)$).	sdAEx	—
Standard deviation of delta time between consecutive local extrema (*sd*(*dt*_*ex*_)).	sdDtEx	—

The feature extraction module allows to selectively include subsets of the listed features in the feature vector, depending on the configuration of the extraction step within the hosting process. In order to extract specific properties from the output of distinct preprocessing stages, e.g., from high-pass filtered, low-pass filtered, or unfiltered EEG data, it is possible to include the same feature extraction step multiple times within a single process, with each instance receiving a different input data set.

#### 8.2.2. Frequency Domain Features

Events of interest can be analyzed with regard to their characteristics in the spectral representation. Frequency domain analysis in our software framework is currently limited to a static spectral decomposition. We use Fast Fourier Transform with a Tukey window and α = 0.5, the ratio of the cosine-tapered length of the full window, applied upfront. The resulting magnitude values are aggregated into frequency sub-bands (bins) of defined width. Lower and upper frequency limits, as well as the sub-band width can be specified. The calibrated magnitude of each sub-band is added to the feature vector as a separate feature. Optionally, the generated spectrum can be normalized to the value range [0..1]. The computed scaling factor is considered as a further feature in this case.

A more elaborate spectral analysis could generate further potentially useful and distinctive features, such as the spectral centroid (Blanco et al., 2010; Matsumoto et al., 2013; Pearce et al., 2013) of the segment. In particular, a time-frequency analysis could yield interesting details about the development of the signal's spectrum throughout the event. Systematic tests would be necessary to assess the added value of these properties in addition to the static spectrum.

#### 8.2.3. Feature Subset Selection

Depending on the number of observed input data properties and their numerical representations, feature extraction may result in feature vectors of considerable length, i.e., in high-dimensional feature spaces. Not all of these elements, particularly of high-dimensional feature vectors, contribute equally to the quality of the constructed predictive model. The longer a feature vector, the higher the chance that some features convey redundant information or are insignificant or even irrelevant with regard to the targeted classification (Dash and Liu, 1997; Guyon and Elisseeff, 2003). Obviously, this may significantly increase computational cost in terms of both, memory usage and CPU time, for no benefit.

The second aspect mandating dimensionality reduction is the risk of poor generalizability of the resulting model in case the extracted features are adapted too closely to the training data set. This problem is known as “overfitting” (Shalev-Shwartz and Ben-David, 2014). Generally, the training data samples are assumed to be selected independently and identically distributed from and, thus, to be representative for the underlying distribution (universe). In that case the ideal strategy would be to minimize the empirical error, i.e., the error in classifying the training data set [“Empirical Risk Minimization,” see Shalev-Shwartz and Ben-David (2014)]. Since EEG recordings differ from patient to patient and are influenced by a number of factors, such as circadian rhythm, received medication, activity, or exogenous artifacts, this assumption is not valid for the HFO classification case. The better the classifier performs on the training data set, the higher the risk of overfitting and failing on previously unseen recordings, i.e. the “true error” increases with a decreasing empirical error. Reducing the number of features according to a suitable algorithm may also decrease the risk of overfitting by reducing the susceptibility of the constructed model to (potentially exceptional) peculiarities of the training data set (Nilsson, 1998).

A third reason calling for a reduction of feature vector length is an improved understandability of how characteristics of the data are related to the classification outcome. Knowing which properties are the most influential and discriminating may be of interest also for the advancement of analytical HFO detection algorithms.

Feature subset selection addresses the issue of finding the smallest subset of features that yields the best classification performance. This problem raises two questions: how to select a particular subset? And how to assess its performance? The conceptually simplest solution is to minimize the true error using all possible combinations of features and to validate each combination using samples not in the training data set (Guyon and Elisseeff, 2003). Considering the exponential complexity requiring 2^|*F*|^ iterations, with |*F*| the dimension of the feature space, this approach is computationally not feasible.

The algorithm implemented in our SVM classifier module iteratively partitions the provided training data set into a training and a validation subset (resampling with replacement) and assesses the predictive power of selected feature subsets by creating a new model in each iteration. Technically, this so-called “wrapper” method is agnostic with respect to the particular type of machine learner and could be encapsulated in a dedicated module in order to reuse it with potential future classifiers. The encapsulating procedure of the feature subset selector is depicted in Figure S7. The procedure receives the full set of features and the training data set as input items. In an outer loop the training data set is randomly shuffled and partitioned into a training subset and a validation subset (“hold-out” samples) using the GSL implementation of the MT19937 random number generator (Galassi et al., 2017).

Subsequently, ANOVA is performed separately on each feature in order to reorder them according to their discriminative power with regard to the classes to be distinguished (ripple, fast ripple, artifact). ANOVA maintains the functional dependency between data and labels and, thus, satisfies this requirement for a suitable variable ranking function (see Song et al., 2012). To avoid a bias by the validation subset, only the training subset is considered in this step. The inner loop, detailed in Figure S8, derives a (locally) optimized subset of features based on the reordered set of features and the disjoint training and validation data subsets. The resulting feature subsets of all iterations of this procedure are aggregated into a feature frequency histogramme. The number of iterations of this outer loop depends on the configured ratio (*r*) of the validation subset size with respect to the total training set size and is calculated as *n* = ⌊1/*r*⌋. The final step of the procedure generates an optimized feature subset using only those features that appeared in at least two locally optimized feature subsets.

The inner loop (Figure S8) uses the “greedy forward selection” algorithm, also referred to as “hill climbing” (Guyon and Elisseeff, 2003), to create a locally optimized subset of features on the basis of the provided training and validation data subsets. The algorithm iteratively adds features, taken from the ordered input feature vector, to the (initially empty) local feature set. This local feature set is used for model generation, using the training subset, and validation, using the validation subset. After each iteration, the feature that resulted in the highest improvement of classification accuracy is permanently added to the local feature set. The loop terminates as soon as no improvement can be made by adding further features. Adding only features that improve the outcome also reduces redundancy.

As the input feature vector is ordered according to the discriminative power of the features, the algorithm tends to converge after a small number of iterations. A further speedup is achieved by removing from the ordered feature set all features that resulted in a classification accuracy $a_{f}<\bar{a}-sd\left(a\right)$ when added, causing them to be ignored in subsequent iterations.

Here, we provide an example based on 19 invasive recordings with macro electrodes (Montreal reference data set) and a sampling rate of 2,000 samples/s. The total number of events was 11434 (avg. 600 events/recording), with a total duration of events of 80,5426 ms (equivalent to 161,0852 samples). The average event duration was 70.5 ms (141 samples), standard deviation of 43.9 ms and median 62 ms. The selection starts with 85 spectral features (i.e., spectral bins). The algorithm proceeds as follows:

- Feature node 1: 11434 instances, 9 features (on high pass filtered data)
- Feature node 2: 11434 instances, 6 features (on low pass filtered data)
- MgpActClsSVM: concatenated 100 features from 3 feature nodes for each of 11434 instances
- MgpActClsSVM: random number generator
- MgpActClsSVM: runs: 10, training set size: 10291, validation set size: 1143, classes: 3
- MgpActClsSVM: Instance count: class[ [EOI] ] = 1379
- MgpActClsSVM: Instance count: class[ [F] ] = 688
- MgpActClsSVM: Instance count: class[ [R] ] = 9367
- […]
- All features that occurred (i.e. were part of the computed subset) at least in 2 of the performed 10 runs were selected for the final subset:
  - MgpActClsSVM: Feature vector: len: 6: [42 43 90 91 93 95 ]
  - MgpActClsSVM: Avg accuracy: 0.973561

The execution time for feature subset selection from 100 to 6 features was in total 137 minutes on a Apple Mac Pro “Eight Core” 2.66, 16 GB RAM, Solid-state disk.

#### 8.2.4. Feature Scaling

Having large and varying ranges of values for different features may negatively affect the classification performance, as those features with the largest value ranges may govern the model generation process and may be a source of overfitting the model to the training set (Lin, 2015). This is of particular concern in geometrical machine learning models. A commonly used technique to mitigate this problem is to scale (or normalize) the features to some common value range (Nilsson, 1998): $x^{\prime }=m+\left(\left(x-x_{min}\right)\left(M-m\right)\right)/\left(x_{max}-x_{min}\right)$, with *x*_*min*_ and *x*_*max*_ the minimum and maximum of the original range and *m* and *M* the normalized minimum and maximum (setting *m* = 0 and *M* = 1 is termed “unity-based normalization”). The implemented SVM classifier module allows to specify *m* and *M*.

During training, the SVM module derives the scaling factors for each feature within each iteration of the outer feature subset selection loop (Figure S7), considering the random training data subset only. Feature scaling is then performed for all samples using the calculated factors. Before concluding a learning process and building the final prediction model, feature scaling is re-performed using scaling factors calculation over the entire training set.

#### 8.2.5. Parameter Optimization

SVM technique partitions the instance (sample) space by a hyperplane (the class or decision boundary) that is defined by its support vectors (those samples of the training set that are closest to the boundary) and the “margin” between hyperplane and support vectors. Since HFO classification involves three classes, ripples, fast ripples, and artifacts, the problem is internally broken down in LIBSVM into a set of three models (*n*(*n*−1)/2 for *n* classes), each distinguishing between two classes (Chang and Lin, 2011).

According to Cortes and Vapnik (1995), what they term “soft margin hyperplane” can be found by minimizing the functional $\frac{1}{2}w^{T}w+C\underset{l}{\sum }\xi_{i}$, with *w*^*T*^ the transpose of the (weight) vector *w* defining the hyperplane and $\underset{l}{\sum }\xi_{i}$ the sum of training errors. A training error ξ_*i*_ is defined such that $y_{i}\left(w^{T}\cdot \phi \left(x_{i}\right)+b\right)\geq 1-\xi_{i}$ with ξ_*i*_>0, *x*_*i*_ an element of the training set, ϕ a feature space mapping function (see below), *y*_*i*_ the respective class label (1 or −1), and *b*, bias, a scalar. (The optimal class-separating hyperplane would imply ξ_*i*_ = 0 for all samples of the training set, while 0 < ξ_*i*_ < 1 results in a margin violation and for ξ_*i*_>1 the corresponding sample *x*_*i*_ is misclassified Zisserman, 2015.)

LIBSVM allows to adjust the regularization (or misclassification penalty) parameter *C* which controls the emphasis that is put on misclassifications during training, i.e., how hard the learner shall try to avoid misclassifications of training samples. A smaller *C* results in a wider margin and a reduced risk of overfitting the model to the training data, at the cost of possibly misclassifying some training samples.

Support vector machines can transfer the feature vectors into a higher-dimensional feature space in case the data is not separable in the original feature space, enabling non-linear decision boundaries in the original feature space. This process requires the computation of the inner product of the mapping function ϕ: 〈ϕ(*x*_*i*_), ϕ(*x*)〉 which can be efficiently calculated (without knowing ϕ) using a suitable “kernel function” *K*(*x*_*i*_, *x*) with *x*_*i*_ a single vector of the training set and *x* the sample under test (Jordan, 2004).

The implemented SVM classifier module configures LIBSVM to use the Gaussian radial basis function (RBF) as a kernel function. It is defined as $K\left(x_{i},x\right)=e^{-\gamma ||x_{i}-x|{|}^{2}}$ with γ≥0 the weight associated with the distance ||*x*_*i*_−*x*||. *K*(*x*_*i*_, *x*), thus, gives a weighted measure of distance or “closeness” of the values *x*_*i*_ and *x* and converges toward 0 for increasing values of γ and toward 1 for decreasing values (Shalev-Shwartz and Ben-David, 2014). From a simplifying perspective, this metric can be seen as a measure of complexity of the hyperplane or “peakedness” toward its support vectors. A large γ trims the model more tightly to the support vectors increasing the risk of overfitting, while a small γ may not be able to reflect the complexity of the data (Keerthi and Lin, 2003; Ansari and Ahmadi-Nedushan, 2016). LIBSVM allows to adjust γ to tune the predictive model as required.

Since the parameters *C* and γ mutually influence each other the SVM classifier module optimizes them in a grid (quadratic loop) fashion. The configurable ranges for both parameters are exhaustively probed, computing the accuracy via cross-validation over the training set for each combination of values. A clear disadvantage of this method is the quadratic complexity *O*(*n*^2^). More elaborate parameter optimization algorithms, such as proposed by Keerthi et al. (2007), could be considered in the future in order to reduce the computational cost.

### 8.3. SVM-Based Detection and Classification Process

The flow of data items, from the provision of raw EEG input data to the output of classified HFO markers, and the life-cycles of necessary temporary data nodes depend on the configuration of the process. Figure S9 illustrates the movement of information within a HFO detection process based on SVM classification. The process comprises four stages, arranged horizontally from left to right in the figure, which are traversed sequentially (see also Figure 7).

- In the **preprocessing** stage a “FIR filter” activity creates a high-pass and a low-pass filtered version of the raw EEG recording it receives as input.
- The “RMS-based event detection” activity in the **EOI detection** stage receives the high-pass filtered EEG and analyses it for occurrences of potential high-frequency oscillations which are compiled into a list of event markers.
- The **feature extraction** stage hosts two activities. The “Frequency-domain feature extraction” step uses the EOI list generated by the detector to create a feature vector from the normalized spectral decomposition of the high-pass filtered EEG data segment corresponding to each listed event. The second activity, “Time domain feature extraction,” extracts morphological properties from both, the low-pass and the high-pass filtered versions of the EEG recording. Again, the EOI list defines the data segments to be analyzed.
- The “classification” stage is the final stage of the process. It hosts an instance of the SVM-based classifier which relabels the events in the EOI list into “ripple,” “fast ripple,” and optionally “artifact,” based on the aggregated feature vector associated with each event. In validation mode (see below) the activity generates a classification performance report, informing about the achieved level of accuracy.

#### 8.3.1. SVM Classifier Operational Modes

The implemented SVM classifier module can be operated in three distinct modes that depend on the context in which the hosting process is executed.

1. In **learning** mode, the classifier uses the input EEG data to generate a predictive model based on the labeled (pre-classified) sample events, i.e., the training set, and persistently stores the model for subsequent validation and classification runs. The process of model generation includes the feature subset selection and parameter optimization activities discussed earlier.
2. The classifier's **validation** mode is invoked in order to assess the classification performance of a previously generated predictive model. The classifier processes potential HFO occurrences provided by the detector, classifies them, and compares the classification results with the labeled events that are contained in the original EEG input files. Classification performance is expressed in terms of sensitivity or true positive rate, precision, false negative rate, false discovery rate, and F1 score.
3. In **classification** mode the classifier applies a previously generated predictive model to classify the list of events associated with the provided EEG input recording.

## 9. Usability

Whether a software system is considered useful and appropriate not only depends on the set of implemented functions but is largely related to intuitiveness and comprehensibility of the steps and actions that have to be performed in order to execute a particular function or to achieve a certain goal.

In the case of the implemented HFO detection framework the two targeted groups of users, clinical EEG specialists and neuroscientific staff, have to be considered, who use the system for different purposes and with distinct sets of requirements. While the primary interest of the EEG specialist is to analyse and comprehensively report on HFO occurrence and characteristics within an EEG recording, the neuroscientist uses the system to analyse and evaluate existing approaches, or to develop new HFO detection and classification strategies. The operational concept shall accommodate both scenarios by making the relevant operations available to the EEG specialist in a straight-forward and familiar way, and at the same time providing the required level of adaptability and extensibility to the neuroscientist.

### 9.1. Visual Reviewing Tools

**Figure 8:**
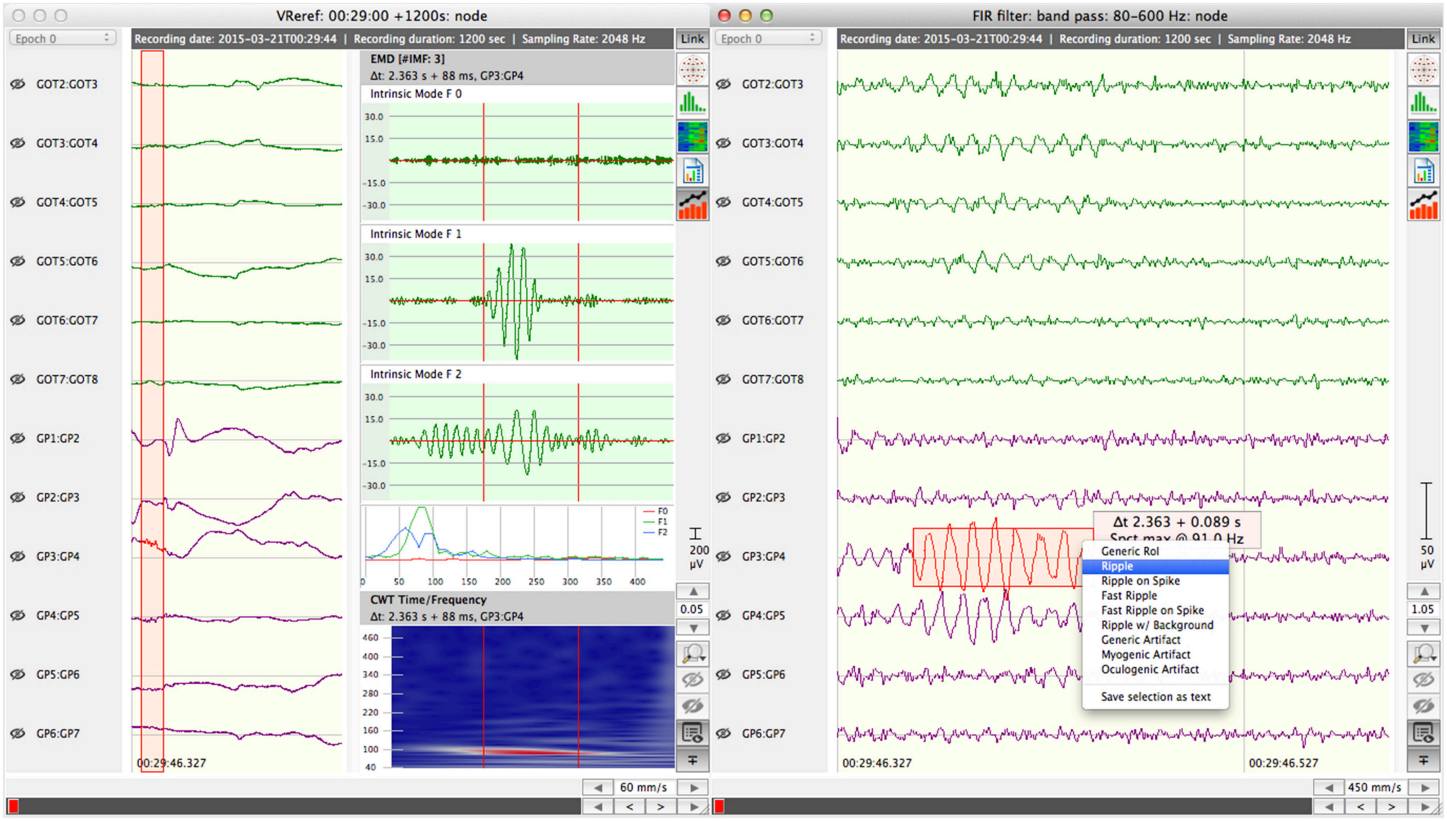
Compacted view of synchronized waveform display. Left window: standard scaling (60 mm/s, 25 mm/mV). Display of empirical mode decomposition and time-spectral analysis of marked segment (red). Right window: amplified waveform (450 mm/s, 400 mm/mV) with marked segment (red) matching the marking in the left window.

Visual review of raw or filtered EEG waveforms is still the most important analysis technique in daily clinical routine. The developed software framework adopts traditional EEG viewer functions, supported by a comprehensive set of tools that provide alternative views of the data, such as time-frequency representation, with focus on visual recognition of high-frequency oscillations. Even though the system is centered around automated signal analysis, visual reviewing tools are important in order to analyse and understand the morphological and spectral characteristics of valid HFOs and their distinction from artifacts, an essential precondition for the development and optimization of well-performing automated detection strategies. Moreover, the definition of an expert-validated “ground truth,” i.e., assembling an ideally large set of HFO events that is agreed upon after visual analysis of representative sample data, which is the basis for assessing the performance of automated detection algorithms, requires the availability of suitable visual tools. A typical waveform display is shown in Figure 8 in compact form. The screen is split into two synchronized display windows. The left window reproduces the original zero-mean corrected EEG signal in standard scaling with a time resolution of 60 mm/s and an amplitude scaling of 25 mm/mV. The right window shows a high-pass filtered copy of the same EEG recording, starting from the same point in time but with increased temporal resolution and amplified voltage scale (450 mm/s, 400 mm/mV). Mouse or cursor keys can be used to scroll through the recording with both display windows maintaining their synchronicity. Temporal and amplitude scalings can be adjusted independently for each active display window. A number of scaling presets cover suitable adjustments for different levels of detail of data display.

Whenever the user marks a potential HFO event in the right window, a small overlay window reports the key parameters (time offset, duration, and peak frequency) of the marked data and a pop-up menu allows to tentatively classify the selected segment. A later reclassification of segments is possible. The display window on the left projects the selection in proper time and amplitude scaling onto the original data. An overlay “analysis” pane that contains a configurable set of alternative representations of the selected segment can be enabled for each of the display windows. The analysis pane depicted in Figure 8 shows the first three intrinsic mode functions of an empirical mode decomposition as well as the time-frequency representation of the segment plus an equally-sized environment before and after the selection. Red vertical lines demarcate the selected segment (middle) and the surrounding environment (left, right).

In addition, the system's visual review tools currently implement the following views that can be added to the analysis pane:

- Spectral decompositions of the selected segment and the selected segment including its environment. This view allows to estimate which frequencies are more dominant in the segment in comparison to its direct temporal environment, and, thus, to identify specific distributions in spectral power.
- The discrete wavelet packet decomposition (DWPD) extracts the signal power over the segment's duration using discrete time and frequency steps. Similar to the continuous wavelet transform it depicts the time/frequency distribution of signal power.
- The short-term Fourier transform (Gabor transform) decomposes the non-stationary signal into small segments which are considered stationary. FFT (fast Fourier transform) is separately applied to each segment. It is a faster alternative to the continuous wavelet transform, however, its temporal resolution needs to be traded off against its frequency resolution by adjusting the partitioning window function.

**Figure 9:**
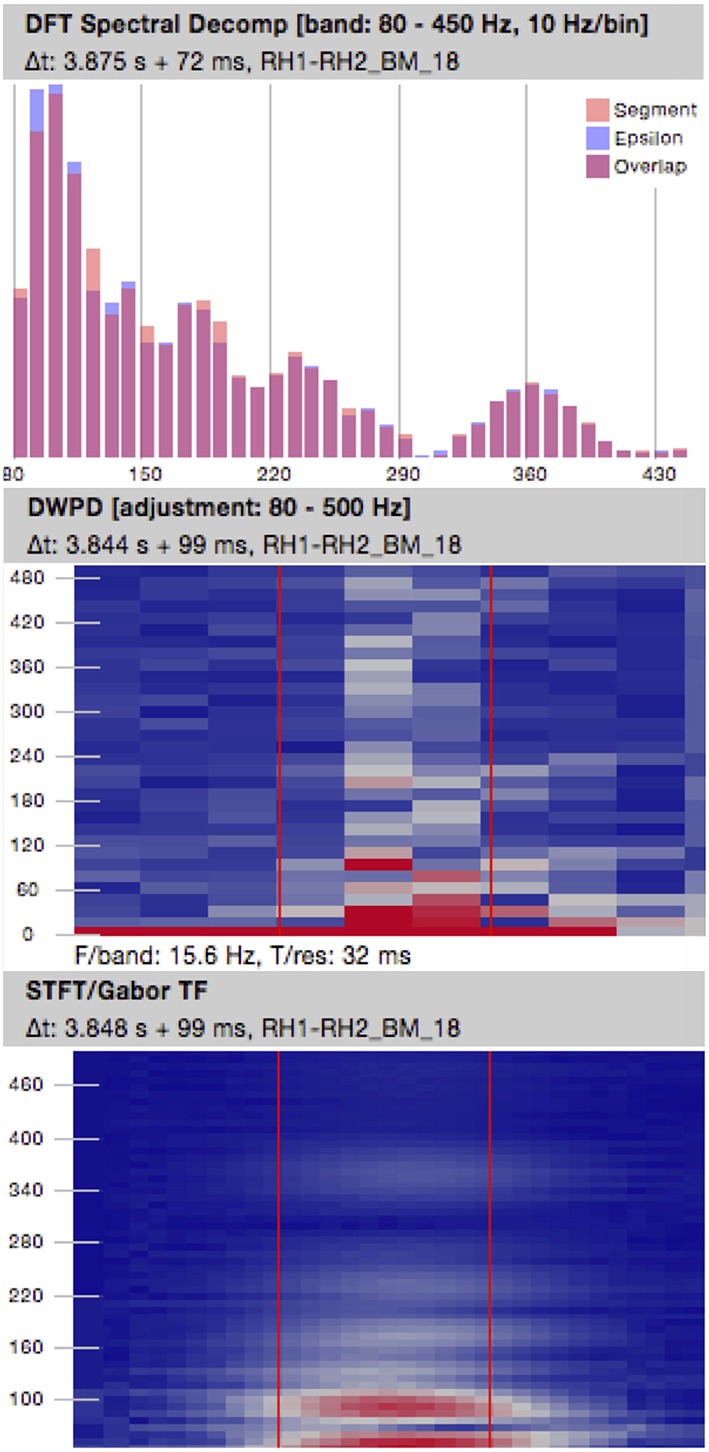
Additional segment analysis views. **(Left)** Spectral decompositions of selected segment (orange), selected segment plus environment (blue). Purple bins represent the overlap. Bin width 10 Hz. **(Middle)** Discrete wavelet packet decomposition, time vs. frequency. **(Right)** Short-term Fourier transform (Gabor transform), time vs. frequency. **(Middle, Right)** Power increases from dark blue to red. Red vertical lines demarcate the selected segment and environment.

Figure 9 illustrates these analysis views by example of a 100 ms segment of a depth electrode signal.

### 9.2. Signal Preprocessing

Virtually all applications, including the visual detection of HFO events, require the raw recordings to be preprocessed in order to reduce the data to the relevant spectral or temporal components. The following filtering and segmentation mechanisms are implemented in our software system:

#### 9.2.1. Filtering

A FIR (finite impulse response) filter allows to reduce the bandwidth of a recorded signal and to extract particular frequency bands. The filter is based on a windowed-Sinc function (Smith, 1998) and uses forward/inverse FFT to reduce the computational cost. The filter allows to optionally specify a lower and/or upper frequency limit and, thus, is able to operate as a low-pass, high-pass, and band-pass filter. As an additional configurable option, the resulting filter kernel may be convolved with itself which leads to a further improvement of its stop-band attenuation (< −145dB typically).

In a study using simulated and real EEG data Bénar and colleagues (Bénar et al., 2010) discussed the problem that FIR filters may introduce “false ripples,” oscillatory artifacts that could be mistaken for real HFO events, when filtering signals that occupy a very wide or even the entire frequency spectrum (band-unlimited signals). The less sinusoidal and the steeper a signal transient the wider is typically its spectral coverage. In the extreme case of a unit impulse the signal's energy is equally spread across the entire spectrum. Filtering a signal that has similar properties, as may be the case with sharp epileptic spikes, may result in an output signal closely resembling the impulse response of the filter, which is, in the case of Sinc-based FIR filters, a symmetric short-term oscillatory signal.

Empirical mode decomposition (EMD) is a technique that can be used to extract a particular sub-band from a given signal in replacement of classical filters (Flandrin et al., 2004). Conceptually similar to the Fourier transform (FT), the EMD approach decomposes a signal into its narrow-band constituent functions or modes. In contrast to FT, which is based on a system of sinusoidal basis functions, the EMD is a purely data-driven technique that does not assume any specific inherent function type or template.

The algorithm is executed iteratively and calculates in a first step the envelope that connects the local minima of the signal segment as well as the envelope connecting the local maxima. Most frequently cubic splines are used to interpolate between consecutive local minima or maxima. In a second step the mean of the two envelopes *m*(*t*) = (*env*_*min*_(*t*)+*env*_*max*_(*t*))/2 is subtracted from the original signal resulting in a differential sequence *d*(*t*) = *s*(*t*)−*m*(*t*). These steps are repeated with *d*(*t*) replacing *s*(*t*) in each iteration until the mean of *d* over the processed segment ($1/T\underset{T}{\sum }d\left(t\right)$) is smaller or equal some ϵ (the “stop criterion”). The resulting sequence *d*(*t*) is the first “intrinsic mode function” (IMF) and contains the “highest frequency” components of the input signal. The entire procedure is repeated using *m* as the new input signal. The algorithm terminates after the desired number of IMFs has been calculated or *m* is linear.

The EMD implementation of the software framework allows to generate a contiguous subset of intrinsic mode functions, specified the first and the number of following IMFs to be extracted from an input signal. The stop criterion (ϵ) for a single IMF is defined as the minimum threshold of $\underset{T}{\sum }\left({\left(s\left(t\right)-d\left(t\right)\right)}^{2}/s{\left(t\right)}^{2}\right)$ in our case. Figure S10 depicts the configuration panels for FIR filter and EMD. Utilizing the EMD technique it is crucial to consider that, in contrast to conventional filters, it is not possible to a-priori specify the exact frequency sub-band to be extracted (see Rilling et al., 2003). Rather it is an adaptive mechanism that partitions the input signal into high and low frequency components. The IMF “sifted” from each iteration's input signal is defined as a waveform that has a zero crossing in between each pair of consecutive extrema and a zero (plus ϵ) mean. By definition, the spectrum of each IMF depends on the local input signal and may vary over time.

#### 9.2.2. Segmentation

Many use cases require the analysis of only certain segments of a recording. These segments may be arranged around external triggers or events, or may be defined as particular time windows relative to the start of the recording.

Our software framework supports the extraction of time-based segments from any raw or preprocessed recording. Based on a list of time-window definitions (Figure S11, left) of arbitrary length the segmentation process creates a new data node that contains a single contiguous epoch per time-window (i.e., a third-order matrix node). All data portions apart from the defined segments are skipped.

The definition of segments anchored to external triggers or events requires a hosting event track to be included in a recording and is, thus, possible for supported external EEG data file types only (see section 6.1). The event-based segmentation process scans the input file for trigger or event definitions and presents them in a selection dialog that allows to specify the event/trigger types of interest along with the duration of the segment to be extracted (Figure S11, right). A separate epoch for each event that matches one of the selected types is created as a new two-dimensional matrix which is stored as an element of a third-order matrix node.

### 9.3. Process Configuration

Section 7.2 details the concept of configurable processes as implemented in the software framework. Each process comprises a sequence of activities (processing steps). Most of these activities require a set of parameters that are either pre-defined or need to be chosen by the operator. For that purpose each activity provides a parameter configuration widget (panel) which reports the relevant parameters and allows the modification of their values. In course of process configuration instances of these widgets are aggregated and displayed within a configuration dialog, permitting the operator to navigate within the sequence of activities and coordinate the parameterization of the process (Figure S12, left). The process configuration dialog includes an additional tab “References” which informs about the literature that constitutes the theoretical foundation for the implemented algorithm(s) and which may serve as a directory of sources of detailed information (Figure S13, right).

Once a well working set of parameters has been developed for a particular process the values can be persistently stored. Each stored parameter set is associated with a unique textual identifier that allows to restore and use it in subsequent process runs (Figure S14). An arbitrary number of parameter sets can be stored for each defined process. Execution of a process on the currently active data node is initiated using the current set of parameters as soon as the operation selects the “Ok” button.

### 9.4. SVM Model Generation and Validation

Processes may integrate machine learning techniques to classify tentative HFO events (see section 8). Similar to purely analytical processes, machine learning based processes may require the adjustment of sets of parameters for each of their preprocessing, event detection, feature extraction, and classification steps.

As a major difference to analytical processes the classification step of machine-learning based approaches is based on previously generated and validated predictive models. Typically, these model generation and validation processes are not executed on a single (the currently active) data node, but on a subset of available recordings. A dialog that is presented at the beginning of the process configuration activity allows to define the subset of data nodes to be considered as the training or the validation set (Figure S14, listbox on left side of dialog).

In addition it is necessary to specify the marker types according to which detected events shall be differentiated throughout the learning and validation processes. The listbox on the right-hand side of the dialog depicted in Figure S14 presents the respective selection. The listed marker types are read from the software framework's static configuration file.

The further steps of the parameterization of a machine-learning based process are analogous to analytical processes. After suitably configured preprocessing steps the sequence of activities contains an event detection module which identifies the regions of interest that are subject to feature extraction and analysis in the following steps. The same types of event detectors as for analytical algorithms (e.g., Staba et al., 2002 or Burnos et al., 2014) can be used. Subsequently, the subset of signal properties (features) to be evaluated by the learner/classifier has to be defined. Figure S15 depicts the parameter configuration panels for time domain related (left) and spectrum derived features (right).

Specifically for the model generation and validation processes the correlation in terms of temporal overlap and channel between the detected “events of interest” and the predefined markers contained in the input data nodes is essential. During model generation (learning) these markers are used to relabel the matching events of interest, while in the validation process the markers define the ground truth to validate the predicted labels for the detected events against. The event types to be relabeled and the labels of the reference markers need to be specified along with the minimum amount of temporal overlap between any two predefined and detected events that is required in order to consider them as matching. The parameter configuration dialog integrates a dedicated panel for this purpose (Figure S16, left).

As the final parameterization step of a machine-learning process the parameters for the learner/classifier have to be adjusted. Figure S16, right-hand side, shows the configuration panel implemented by the SVM classifier module. The panel reflects the settings required for feature scaling, feature vector optimization, and regularization and distance weight optimization, as described in detail in section 8. The predictive model resulting from the learning process is stored persistently using the “Model file” name specified in the dialog. Like for analytical processes, parameter sets can be stored and restored for future use.

#### 9.4.1. Model Validation

The parameterization steps of the learning and the validation processes are identical with the exception of the “Model file” name which obviously must refer to an existing model in the validation case. During the validation procedure the selected and pre-marked (ripples, fast ripples) input data nodes are used to assess the classification performance of the previously generated model by

1. determining the subset of automatically detected events of interest that overlap at least to the adjusted percentage of temporal overlap (Figure S16, left) with pre-defined markers contained in the input nodes,
2. and comparing the labels predicted for events in the subset with the original labels attached to the matching pre-defined markers.

Those automatically detected EOIs that are not matching any pre-defined ripple or fast ripple events make up the set of artifactual or “generic” EOIs.

For each detected event a “true positive” (*P*_*T*_) counter is increased for the specific type of event if the prediction result matches the original label. In case the prediction fails two further counting variables are increased, a “false negative” (*N*_*F*_) counter for the original type of event and a “false positive” (*P*_*F*_) counter for predicted type of event. Based on these counters the following basic statistical values are derived:

- Sensitivity or true positive rate (as *P*_*T*_/(*P*_*T*_+*N*_*F*_)),
- Precision or positive predictive value (PPV, as *P*_*T*_/(*P*_*T*_+*P*_*F*_)), and
- False discovery rate (FDR, as *P*_*F*_/(*P*_*T*_+*P*_*F*_)).

The results of a machine-learning model validation process including the calculated statistical indicators are summarized in a validation report as illustrated in Figure S17. The report may serve as a basis for regenerating and optimizing the predictive model toward a particular target.

### 9.5. HFO Activity Reporting

The execution of an HFO detection and classification process leads to the definition of a set of markers representing ripple or fast ripple events of diverse duration which are distributed over time and channels. These markers can be individually reviewed and modified directly in the data node by means of the visual waveform display. In addition, the software generates reports that summarize the results of a detection process. An “HFO Report” represents the collected and statistically processed set of high-frequency events of a single data node.

**Figure 10:**
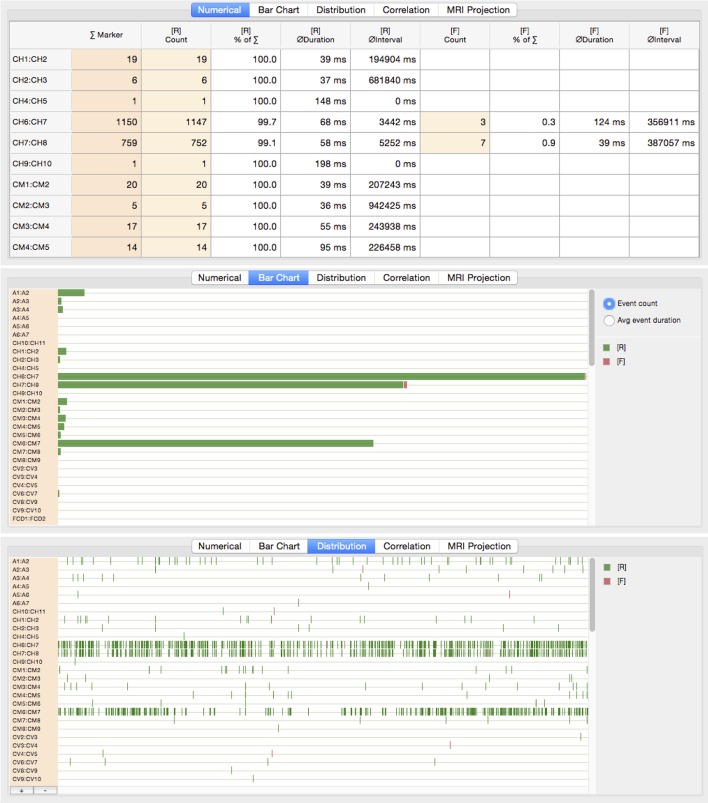
HFO activity reporting modes (example data). **(Top)** Numerical report. **(Middle)** Event frequency per channel and event type. **(Bottom)** Event distribution per channel over time.

Figure 10 depicts the different modes in which the extracted information is visualized in a report: the top window shows a numerical, tabular report of the event types and their essential properties per channel. It contains the number of events per channel, the percentage of events of each type, as well as the average duration and the average inter-event interval for each event type and each channel. The middle window represents the relative number of events of each type for each channel as a bar chart, allowing quick conclusions on the event frequency compared across channels. The bottom window reports on the event occurrence as a two-dimensional distribution map over time and channels. The map facilitates recognition of channels of a high frequency of events as well as time periods of event clusters.

#### 9.5.1. Correlation Assessment

For the purpose of either optimizing the configuration or parameters of a particular HFO detection process or for comparing the results of different detection strategies it is useful to systematically compare selected subsets of HFO events with each other. In most cases, visually identified and marked HFO events will serve as a reference.

**Figure 11:**
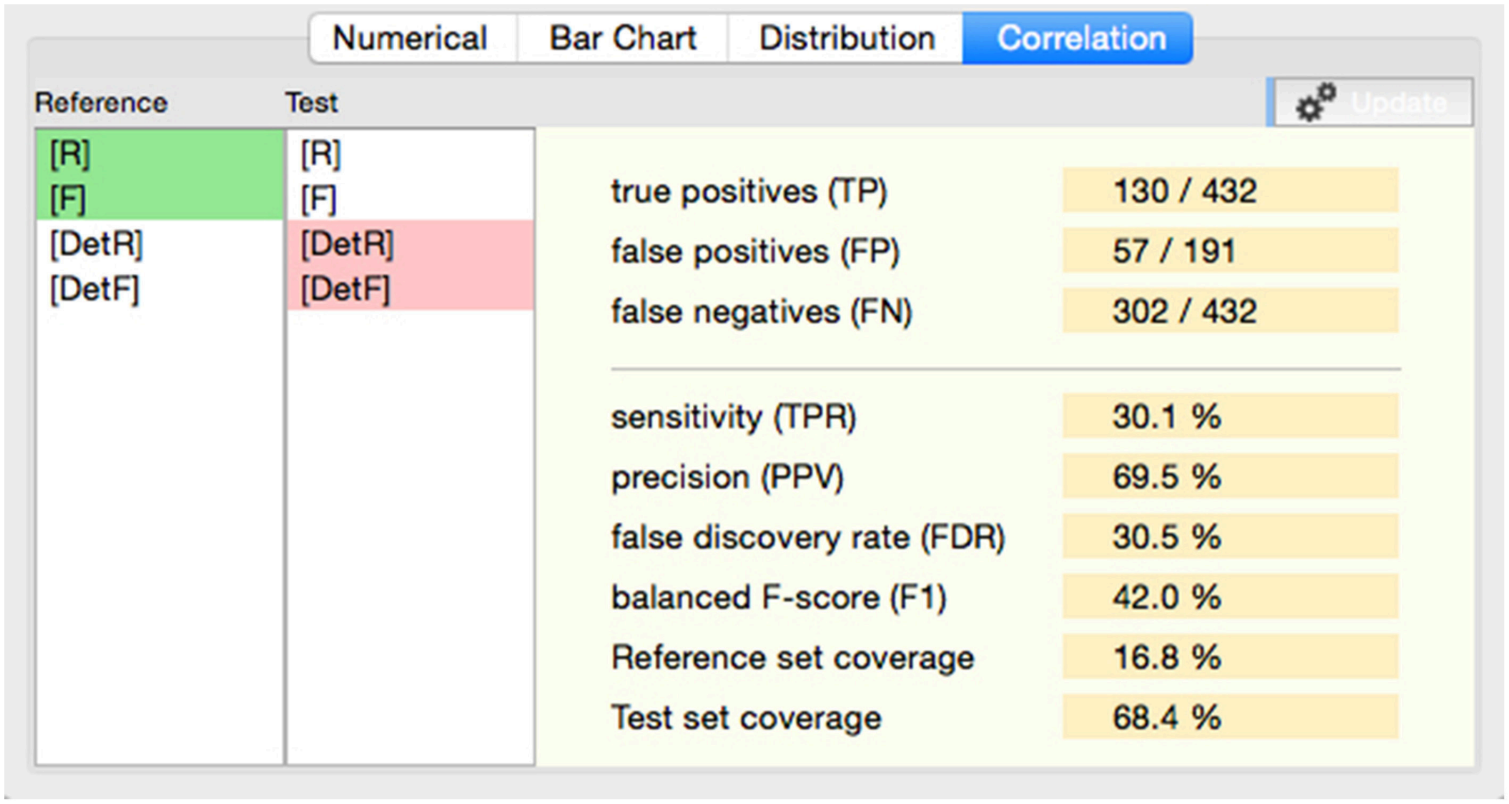
Example of statistical report on temporal and channel-wise correlation of selected types of events. Left column (green): reference subset; right column (red): test subset.

As part of the HFO activity reporting the software framework implements means to determine the types of events that are present in the data node subject to analysis and to partition these types into a reference and a test subset (Figure 11). The results of the correlation assessment are presented in terms of true positives, false positives, and false negatives (misses). Further basic statistical data, i.e. sensitivity, precision, false discovery rate, and balanced F-score are derived from these counters. Additional information about temporal and channel-wise coverage of events of the reference and test sets help to estimate the level of agreement and overlap between the selected subsets.

### 9.6. MRI Integration

Associating detected and classified high-frequency events with the coordinates of their occurrence, both, on the scalp in case of scalp surface electrodes, and on the cortical surface or within brain tissue for intracranial recordings, essentially contributes to the value and usefulness of HFO analysis. Applications of depth electrodes are based on highly individual implementation schemes. The projection of electrode contacts showing high-frequency activity into patient-specific brain MRI image sets significantly enhances the conceivability of the extracted and presented information.

The software framework allows to import and link anatomical MRI image sets available in NIfTI format to any session-defining EEG recording (see also section 6). Associated MRI images are shown in the data nodes dock window at the level of the session's top level data node (Figure S19).

Selecting an imported MRI image from the data nodes list opens the MRI data file in a new window which is partitioned into three planar views, visualizing orthogonal perspectives of the image data aligned with the body axes (longitudinal (z), transversal (x), and sagittal (y) axes) in radiological orientation (Figure S20). The operator can use the keyboard or mouse to navigate through the image planes along any of the axes. Red cross-hairs (cursors) indicate the current position on each of the axes. The three planar views are synchronized so that cursor movements in any of the views causes the others to be updated automatically.

The fourth partition of the MRI visualization window hosts a tabbed sub-window, one tab of which (“Information”) contains informational data, such as file type, value format, or grid scaling factor for each dimension. In addition, two “dial controls” allow to adjust exposure and contrast of the image display (Figure S21, left).

The tab “Volume View” renders a three-dimensional perspective projection of the MRI data (Figure S21, right) using “OpenGL,” a platform-independent high-level graphics library^9^. The translucent image can be rotated and tilted at any angle. A three-axes cross-hair indicates the orientation and position of the viewing planes of the 2D planar images within the 3D projection. By manipulating the observer distance in the translation matrix or by scaling the vertical and horizontal clipping values of the projection matrix the software allows to zoom into the projection or to “step into” the data, projected slice by projected slice.

The 3D rendering and the planar 2D views of the MRI image set are synchronized. Using the keyboard to move the cross-hair in any of the visualizations causes all other views, including the 3D model, to be updated accordingly.

#### 9.6.1. Electrodes Definition

Figures S20, S21 (right) show examples of implanted depth electrodes projected into a patient MRI image. Correct rendering of electrode contacts requires knowledge of electrode types and coordinates. For high-density scalp and depth macro electrode recordings the software framework supports the definition of electrode sets. It derives heuristically the number and types of used electrodes from patterns detected in the contact or (unipolar) channel naming scheme. If contacts are identified purely numerically, a high-density scalp layout is assumed, whereas otherwise the type of electrode is determined by matching identifier prefixes. In this case the system presumes electrodes with equally spaced contacts according the specifications of common electrode types of the following manufacturers:

- DIXI Medical, 2A Route de Pouligney, 25640 Chaudefontaine, France
- Ad-Tech Medical Instrument Corporation, 1901 William Street, Racine, WI 53404, USA

Since both manufacturers provide electrodes with the same number of contacts but possibly different contact spacing, the respective disambiguation is left to the operator. The electrodes set definition procedure leads to the creation of a new data node of type “Electrodes Set” associated with the currently active session (see also section 6.1 and Figure 2).

Selection of the node opens a table of assumed types of electrodes in the set, along with their specifications (length, number of contacts, contacts spacing), identifier prefixes, descriptions, and rendering colors (Figure S22). The table permits the modification of electrode type, naming prefix, and color. In the right-most column of the table an informative description may be added to each electrode.

Defining the contacts' coordinates or electrode trajectories is a manual/visual process in the current implementation. Contact coordinates are specified by their offsets from the origin on the longitudinal, transversal, and sagittal axes. While the location and orientation of a depth electrode is defined by two points, its tip position and its point of ingress into the skull, the position of a surface electrodes is specified by its surface coordinates only.

The “Electrodes Set” panel of the information sub-window, shown in Figure S23, right-hand side, contains a table similar to that of Figure S21, listing all defined electrodes, their prefixes, colors, or descriptions. The columns “Surface Coords” and “Tip Coords” report the pair coordinates associated with each electrode and are initialized to (0; 0; 0). Coordinates are specified by moving the cross-hair visible in the planar views to the respective position and selecting the electrode tip/column point that shall be associated with the current cross-hair position. The planar as well as the 3D views (see Figure S20, right) are updated immediately to reflect the repositioning or reorientation of the electrode.

#### 9.6.2. Overlaying MRI With HFO Activity

**Figure 12:**
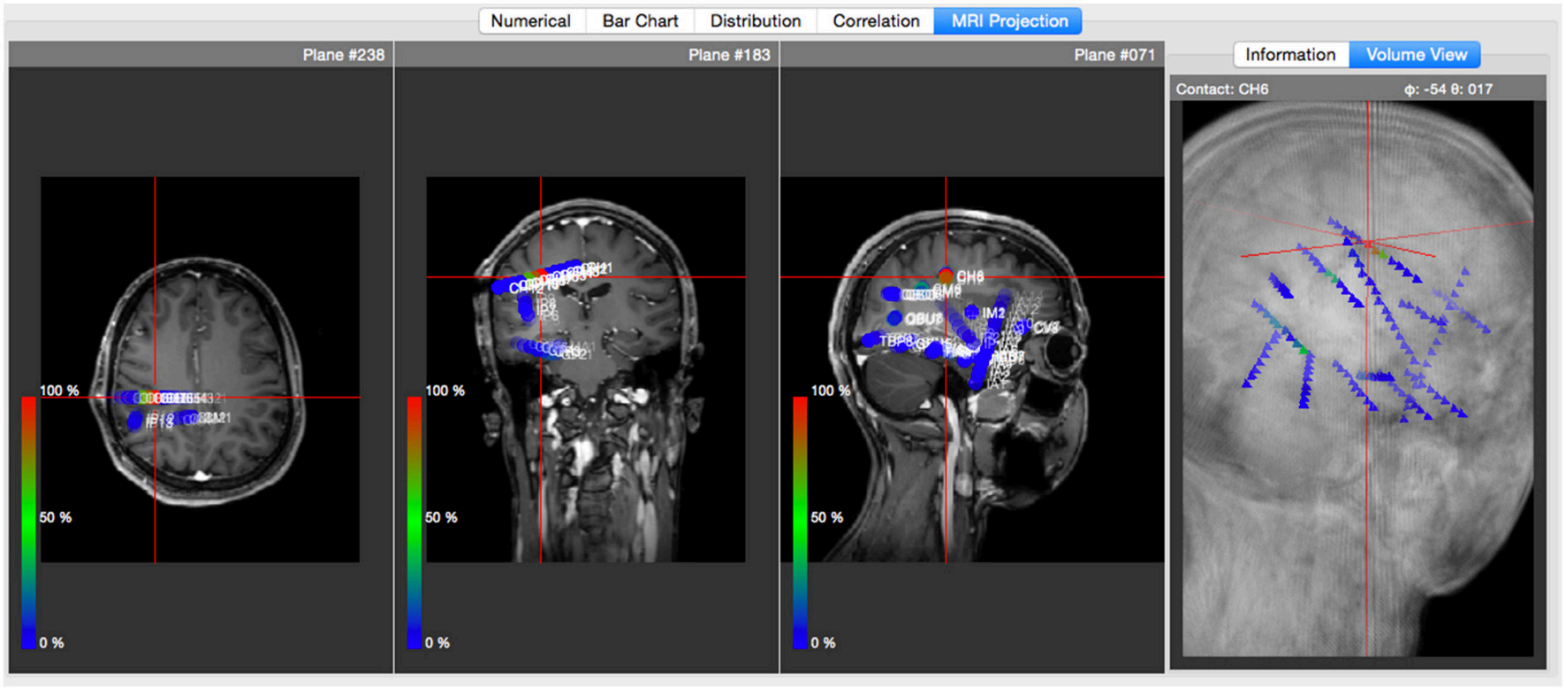
Planar and 3D visualization of spatial distribution of oscillatory events. Downscaled illustration based on example data.

The HFO activity report provides comprehensive information about fast oscillatory events with respect to frequency of occurrence of different types of events, temporal distribution, and distribution across channels. In case the processed recording is associated with a patient MRI image set it is further possible to render the spatial distribution of events into the MRI (Figure 12). A dialog box allows to select the types of events to be considered in the visualization. The frequency of occurrence of selected event types is projected onto the electrodes' contacts using a colored scale reaching from dark blue (lowest frequency of occurrence) to bright red (highest frequency of occurrence).

While the system supports the adjustment of the scale to balance frequency and coloring as well as spatial navigation trough the Image set, the visualization does not convey any information about the temporal distribution of events.

## 10. Efficiency Considerations

In order to keep the software development effort manageable a key design objective was to integrate available software libraries wherever possible and appropriate. This led to the utilization of the following third-party software packages that complement each other in terms of covered functions:

- The “Qt” toolkit^10^ offers a wide range of functions to interact with components of the underlying hardware/operating system in addition to a comprehensive graphical widget set. Qt is available for various operating systems and is a key enabler for the targeted cross-platform interoperability of the software framework.
- The GNU scientific library (gsl)^11^ is a collection of functions for numerical calculations for a large set of areas in mathematics and physics. Our software uses gsl function for descriptive statistical, spline interpolation, random number generation, and permutation functions.
- “FFTW”^12^ is a library that provides function to compute discrete Fourier transforms on real and complex data. All processes within our software that are based on DFT transforms, such as FIR filter, spectral decomposition, Stockwell transform, Hilbert transform, continuous wavelet transform, etc. are based on this library.
- The Support Vector Machine implementation used by the software framework is provided by LIBSVM (Chang and Lin, 2011; Hsu et al., 2016).
- To read and interpret binary MRI image sets in NIfTI format the software makes use of the library “niftilib”^13^.

A different aspect of efficiency is the performance of the software system in terms of computation time required to perform a particular task. Wherever feasible it was attempted to take advantage of current computer hardware architecture, typically providing multiple CPU cores and large amounts of working memory (RAM). Within most activity modules thread-pools are used to process several channels of a recording in parallel, with the number of simultaneously processed channels depending on the number of available CPU cores. When reading large data sets from persistent storage (hard disk, solid-state disk) memory-mapping technique is used that projects the data that physically resides on the storage device into the memory space of the software process. This way, buffer management and preloading of/disposing data are left to the operating system which can optimize the strategy depending on storage type and cumulative requirements of running processes. The use of open source software allows to adapt third-party software to system characteristics if necessary.

Finally, it is of interest to know how sampling rates impact computational time, as varying sources such as scalp vs. microwire recordings use highly differing sampling rates, such as 500 vs. 2 kHz. All of the algorithms analyse the data as a series of samples. If the sampling rate is doubled, this implies that the double amount of samples needs to be processed. This is the same for the analytical algorithms as well as for the feature-providing modules, which extract characteristics from the signals. The extent to which the sampling rate affects computing time depends however on the complexity of the algorithm. In O-notation we could say, O(n) with n being the number of samples or the sampling rate. Thus, if the algorithm has a complexity of A=O(n), the time needed to compute a result, e.g., a detection, doubles with doubling the sampling rate. In contrast, algorithms with *A* = *O*(*n*^2^) would have a four times higher computing time when the sampling rate is doubled. Thus, it depends on the complexity of the algorithm to what extent computing time increases with the sampling rate.

## 11. Discussion

MEEGIPS is a user-friendly software framework with a powerful GUI that allows its use in research and possibly also in clinical practice, given a future appropriate approval by relevant authorities. If we compare MEEGIPS to other software, it turns out that clinical software such as SystemPLUS Evolution, BESA Epilepsy, or NetStation are focusing on the GUI, offering only narrow applicability to research scenarios that demand interoperability and extensibility. In contrast, the research-driven and Matlab-based toolboxes are - except for RippleLab - lacking a comprehensive GUI. Finally, the efficiency of Matlab-based software is naturally lower, as Matlab-written code is between 9 to 11 times slower than the best C++ executable (Aruoba and Fernández-Villaverde, 2015).

### 11.1. Further Algorithms

As stated in section 7.3.2 the list of predefined processes (Table 2) is far from being exhaustive and could be easily extended. A reconsideration of this list should take into account recent studies. Specifically Roehri et al. (2017), have gained insights into the advantages and disadvantages of algorithms, which might influence future decisions on implementing algorithms.

We did not directly compare the algorithms in the present manuscript, as the purpose was rather to present the software framework and its intended use, not the comparison of the algorithms. A direct comparison of the algorithms based on different kinds of signals (see section 11.2) could give new directions. For example, we implemented SVM as a classifier, but recently deep neural networks become increasingly popular and could advance the field in terms of greater gain in learning. Nevertheless, we must keep in mind that deep neural networks perform only well on huge amounts of training data, which we do not have in terms of a gold standard ground truth of manual markings of HFOs. Sophisticated engineering of data augmentation or learning based on simulated data (Höller et al., 2018) could be part of MEEGIPS in future releases.

It is furthermore of interest to re-think feature design for all algorithms based on recent research achievements. Tamilia et al. (2018) found considerable evidence pointing to spatiotemporal aspects of HFO propagation patterns that could be turned into meaningful features for classification. Especially on the surface it is likely that HFOs occurring over multiple sites without propagation represent rather artifacts, while those who propagate and can be detected only over a small spatial extent could be real HFOs. Another important aspect to be considered is prior knowledge on the probability of occurrence of HFOs in specific brain structures (Guragain et al., 2018). The necessity of including prior knowledge into machine learning is laid down in the well known theorem that there is “no free lunch” (Wolpert and Macready, 1995). Including further information that narrows down the area of search or that helps to appraise the likelihood of an event of being an HFO could be a valid approach for future automated detection modules.

### 11.2. Signals From the Scalp

It is questionable whether the currently available algorithms for automated detection perform equally well on scalp-EEG as on invasive EEG data (Höller et al., 2018). Even though principal methodological considerations on automation-supported HFO detection likewise apply to invasive and scalp recordings, almost all published activities are based on invasively collected data (Höller et al., 2018). The comparatively low signal-to-noise ratio, and a variety of artifacts that do not occur to the same extent in invasive recordings represent particular challenges for scalp EEG. In the MEG HFOs seem to occur quite rare, but they co-localize with HFOs detected in invasive EEG (Papadelis et al., 2016) and in scalp EEG (Pellegrino et al., 2016). Therefore, it is highly warranted to implement an MEG module into MEEGIPS. Recordings with large number of channels such as high-density EEG recordings or MEG require additionally highly efficient algorithms and an implementation that takes care of scalability. MEEGIPS provides an ideal base for the inclusion of an MEG module because of its efficiency considerations and because of its modular architecture. It could be further considered to implement a module into MEEGPIS that allows assessing and visualizing the co-occurrence of HFOs in different signals, that is, invasive EEG, scalp EEG, and MEG.

The potential benefits due to the non-invasiveness of scalp EEG—lower risk, lower costs, the possibility to include larger patient populations with different types of epilepsies as well as to conduct longitudinal studies—are unquestioned. In contrast, it was not examined so far whether the use of high-density EEG or MEG instead of conventional (10–20) systems improves the clinical value of scalp HFOs as biomarkers. The first step that should be taken in order to address this research question is to develop specifically adapted computer-supported detection mechanisms. Moreover, future research needs carefully prepared reference datasets with extensive ground truth in order to address the question whether currently available algorithms or newly developed algorithms perform well for automated HFO detection in scalp EEG. MEEGIPS provides the technical framework for addressing these questions.

### 11.3. Limitations

While the choice of C++ reduces execution time by enabling native execution of the code the determining factor for the computational effort is the complexity of the algorithm. This is beyond the problems that be can be addressed by the software framework. This limitation is rather due to the selection of algorithms, not due to the software *per-se*. Detection of HFOs with certain algorithms might just take long because the design of the algorithm does not scale well. For instance, feature subset selection might take very long when the feature vector is very long and the selection algorithm iterates over many possible combinations of features with cross validation. However, memory management of C/C++ is very efficient, so that MEEGIPS has in these terms advantages over MATLAB based toolboxes. The modular design eases furthermore the future change of the implemented algorithms such that new variants are more efficient than previously published versions. For example, computational time related to feature selection can be reduced by sparse discriminant feature selection approach.

MEEGIPS does not include means for securing and protecting the data, i.e. no built-in strategy for backup and encryption of data is available, so far. However, also other clinical systems typically do not include such modules, as backup is usually done via internal mirroring systems within the networks of the clinics. Likewise, data protection within clinical networks is guaranteed by the firewall that applies to all clinical networks. MEEGIPS is, however, compliant with the demands as it includes no module that would transfer data to public storages, cloud systems, nor does it include built-in reporting to the manufacturer in case of crashes. So, there are no modules that would automatically and/or covertly transfer potentially sensitive data. Moreover, MEEGIPS offers no means of manipulating the raw data, that is, no artificial waveforms can be inserted into the original signal.

## 12. Conclusions

The presented software framework MEEGIPS is the result of a structured software development process. It provides a comprehensive user interface with detailed signal analysis and visualization components that support the visual identification for HFOs. In addition to that, the software design allows the flexible extension by additional components such as new HFO detection algorithms. Processes are composed of processing steps (activities), typically preprocessing (filtering, EMD), events of interest detection, and events of interest classification. Variants (i.e., specific algorithms) of these activities can be combined into new processes by modifying the system's process configuration file. Finally, each activity requires a set of parameters. These parameters can be adapted according to the experience or knowledge of an expert.

The scientific development proceeds on a fast pace and the adaptability to new developments of such a system is of utmost importance.

## Author's Note

The software framework will be made available by the authors, without undue reservation, to any qualified researcher. The email request can be sent to meegips@pmu.ac.at.

## Author Contributions

PH has developed the software including software design, project management, and implementation; moreover, he wrote the documentation for these processes. ET provided clinical guidance during the project and he is co-investigator of the FWF funded project within which the development work was integrated. YH is leading the FWF funded project within which the developmental work was integrated, and has transferred the documentation to a manuscript's framework.

### Conflict of Interest Statement

The authors declare that the research was conducted in the absence of any commercial or financial relationships that could be construed as a potential conflict of interest.

## Footnotes

^1^Micromed S.p.A., 31021 Mogliano Veneto (TV) - ITALY

^2^Electrical Geodesics, Inc., Eugene, OR 97401, USA

^3^BESA GmbH, 82166 Gräfelfing, Germany

^4^Compumedics Germany GmbH, 78224 Singen, Germany

^5^The Mathworks, Natick, MA 01760-2098, USA

^6^https://nifti.nimh.nih.gov

^7^http://www.edfplus.info

^8^Extensible Markup Language, https://www.w3.org/XML/

^9^Open Graphics Library, www.opengl.org

^10^The Qt Company, Bertel Jungin aukio D3A, 02600 Espoo, Finland

^11^https://www.gnu.org/software/gsl/

^12^Developed at MIT by M. Frigo and S. G. Johnson.

^13^http://niftilib.sourceforge.net
