# Supplementary material for: MEEGIPS—A Modular EEG Investigation and Processing System for Visual and Automated Detection of High Frequency Oscillations
Source: Front Neuroinform. 2019 Apr 5;13:20. doi: 10.3389/fninf.2019.00020 (PMC6460903; doi:10.3389/fninf.2019.00020)
Supplement: Supplementary file 1 [file Data_Sheet_1.PDF]

Supplementary Material: MEEGIPS - a Modular EEG Investigation and Processing System for visual and automated detection of high frequency oscillations

1 SUPPLEMENTARY FIGURES

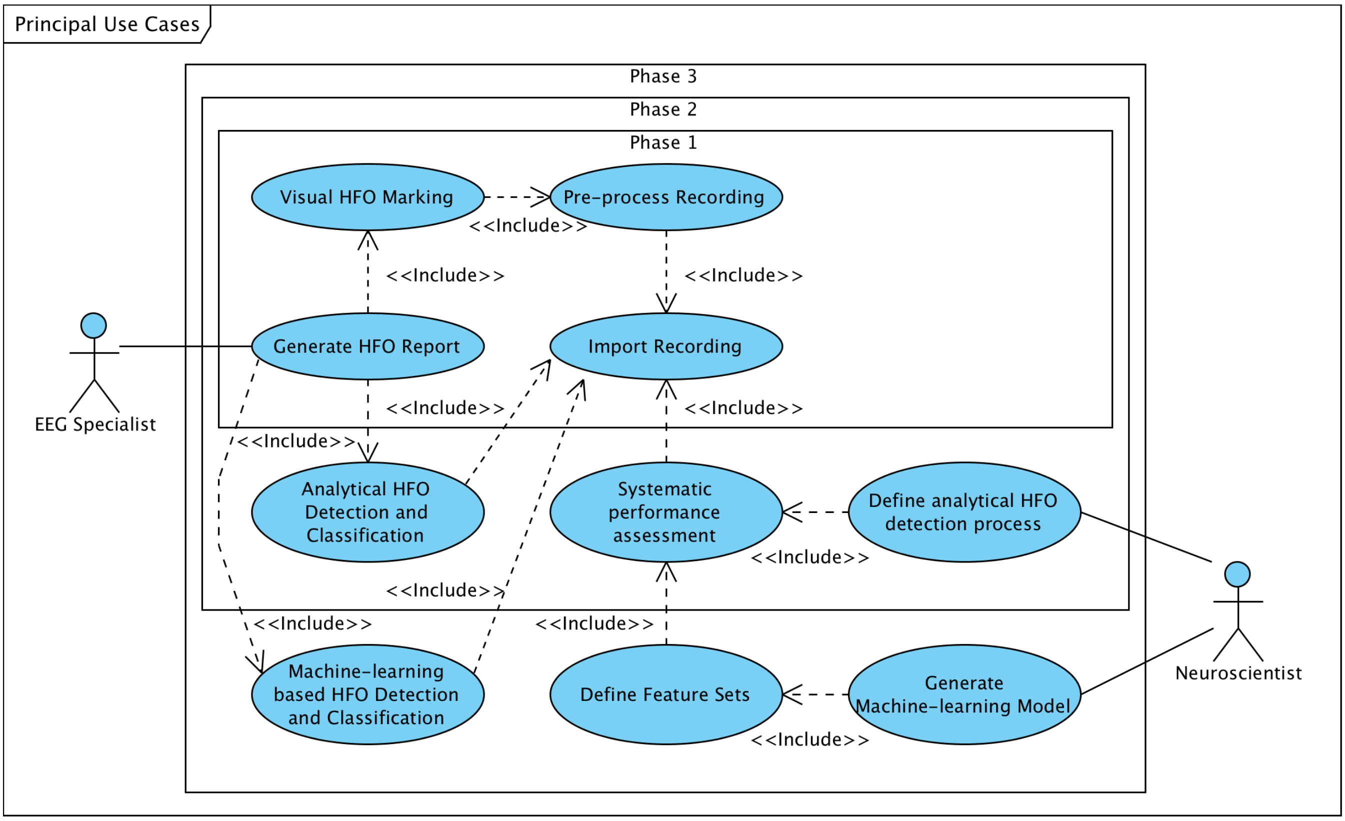

Figure S1. UML Use Case Diagramme.

REFERENCES

Thomschewski, A., Höller, Y., Höller, P., Leis, S., and Trinka, E. (2017). High amplitude EEG motor potential during repetitive foot movement: Possible use and challenges for futuristic BCIs that restore mobility after spinal cord injury. *Front Neurosci* 11, 362

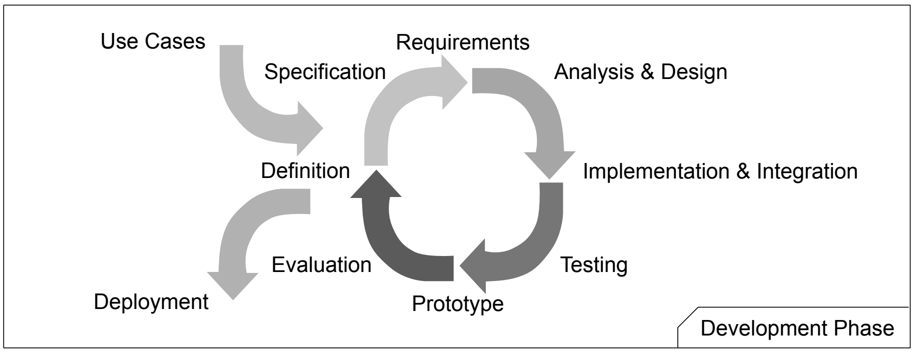

Figure S2. Outline of Incremental Process.

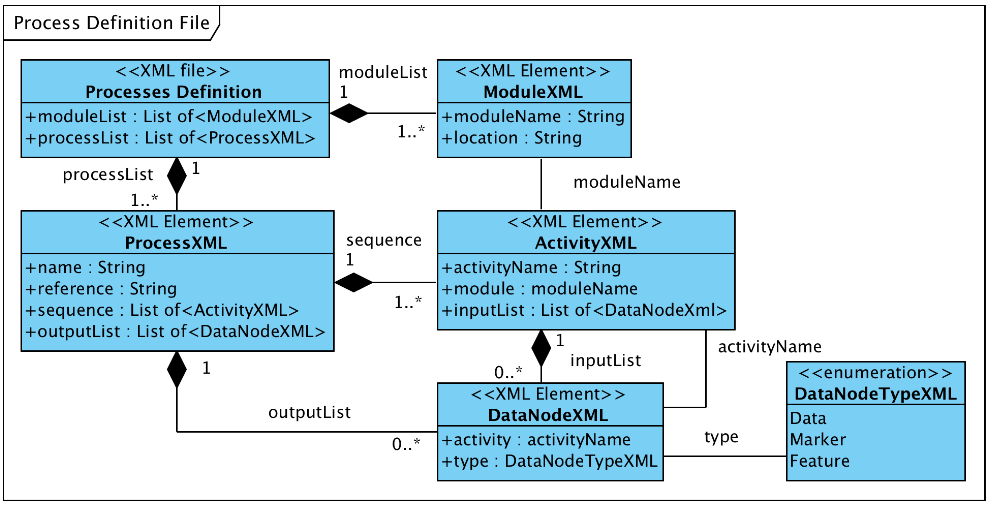

Figure S3. Structure of Process Definition File.

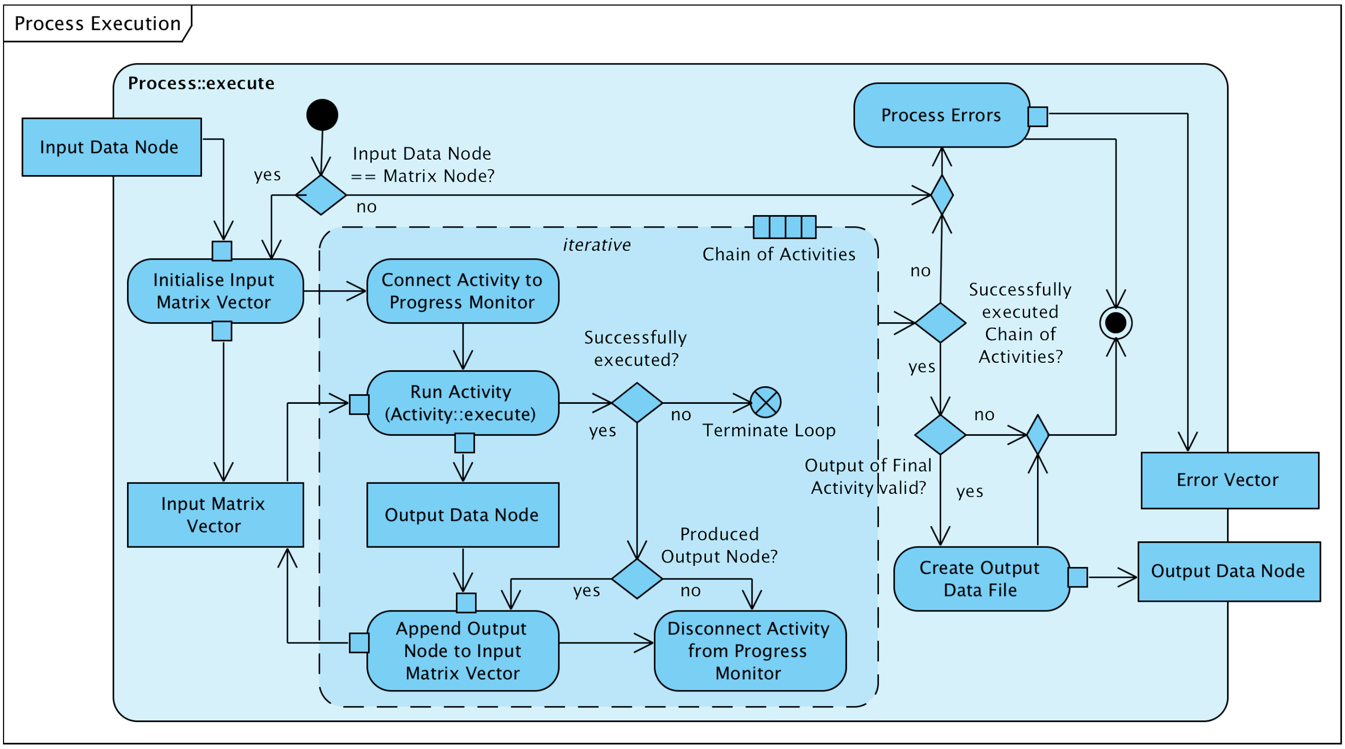

Figure S4. UML Activity Diagramme: Execution of a Process.

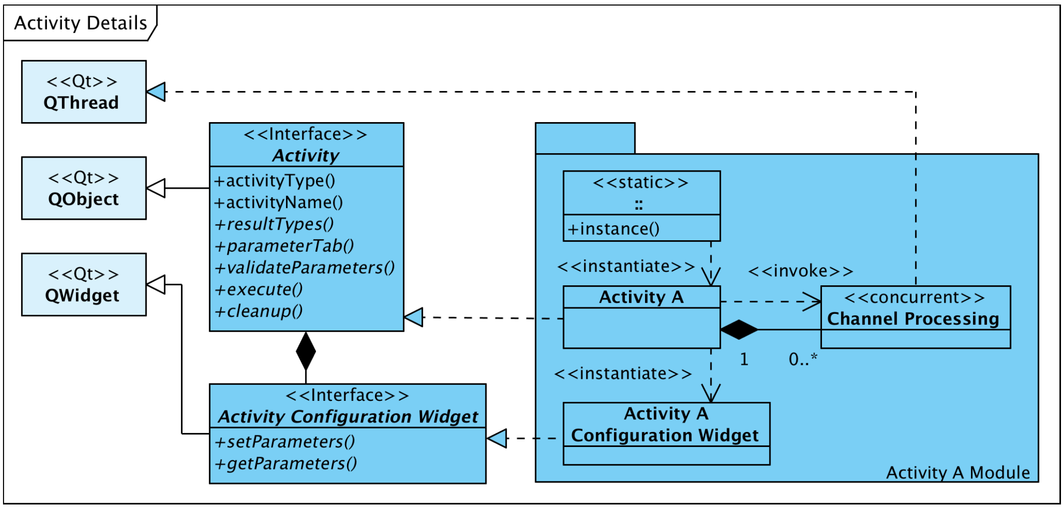

Figure S5. Details of Activity Interface and associated Classes.

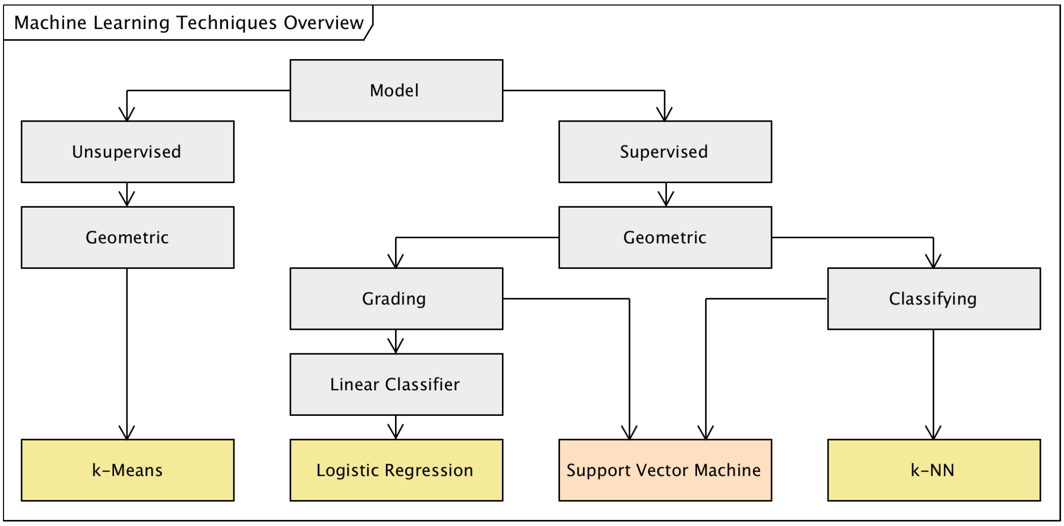

Figure S6. Overview of Machine Learning Techniques used in reviewed Publications.

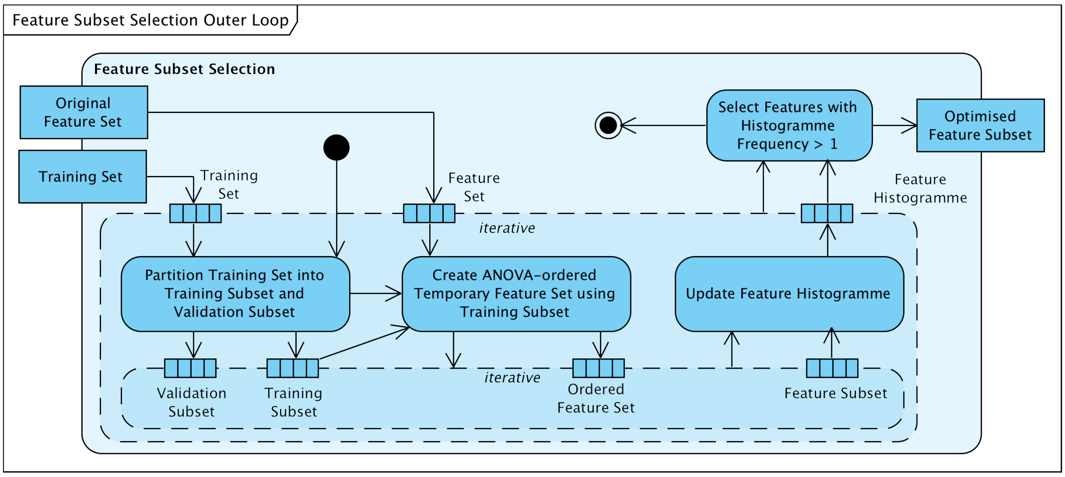

Figure S7. Overview of Feature Subset Selection.

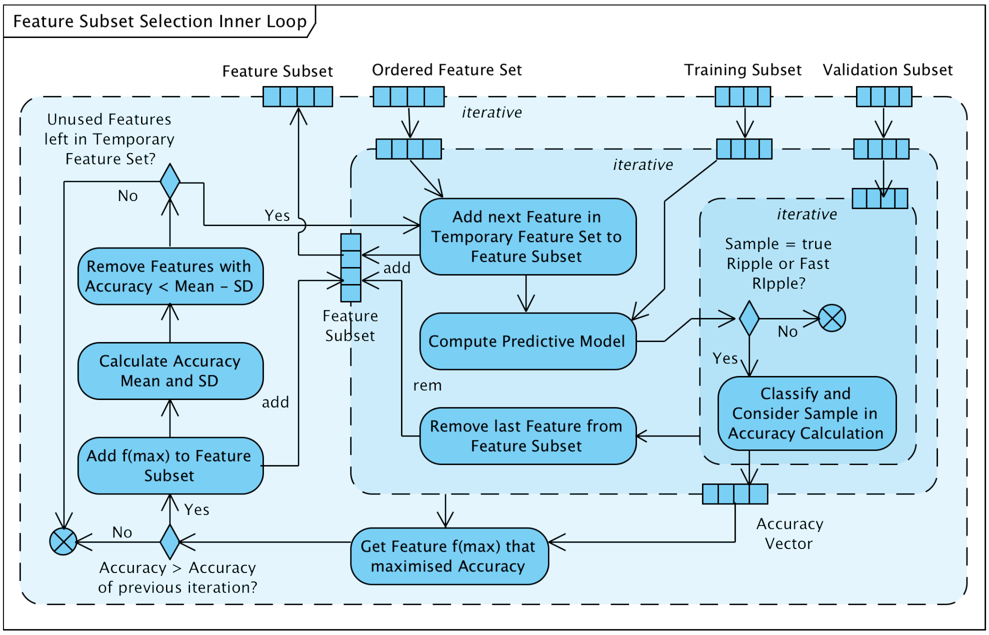

Figure S8. Details of Feature Subset Selection.

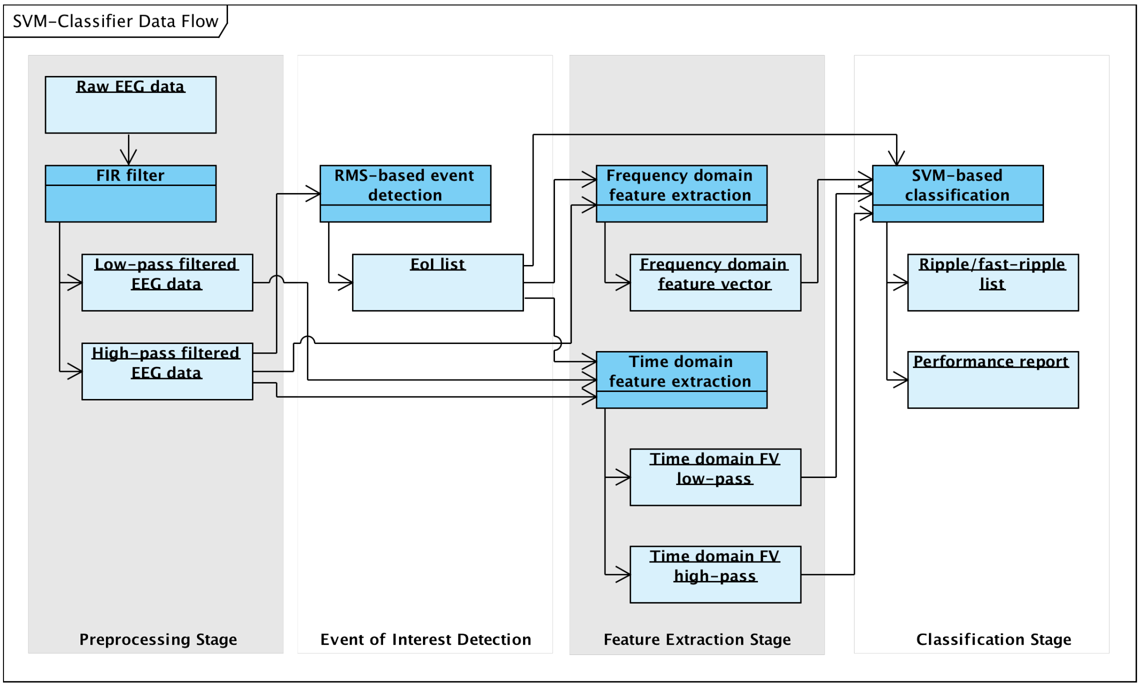

Figure S9. Data Flow Diagramme for SVM-based Classification. Activity Modules depicted in cyan colour, Data Nodes in light blue.

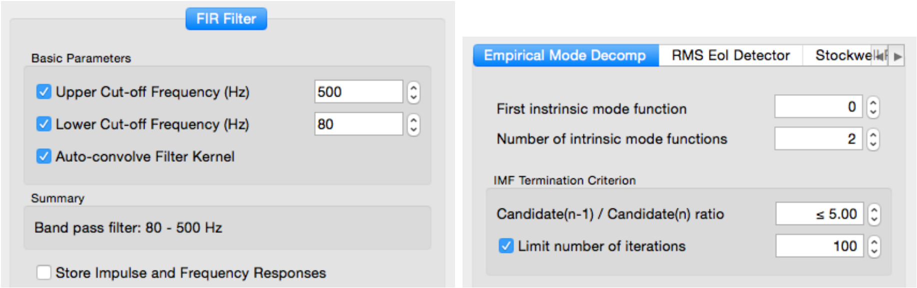

Figure S10. Parameter configuration panels for FIR filter (left) and empirical mode decomposition (right).

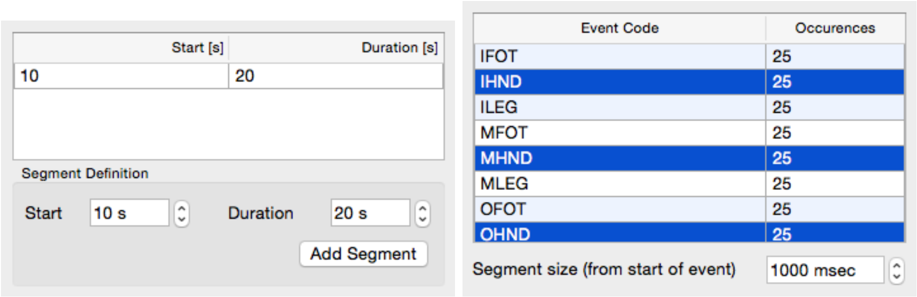

Figure S11. Definition of time-windows for time-based segmentation (left). Selection of event types for event-based segmentation (right): example data from a spinal cord injury study (Thomschewski et al., 2017).

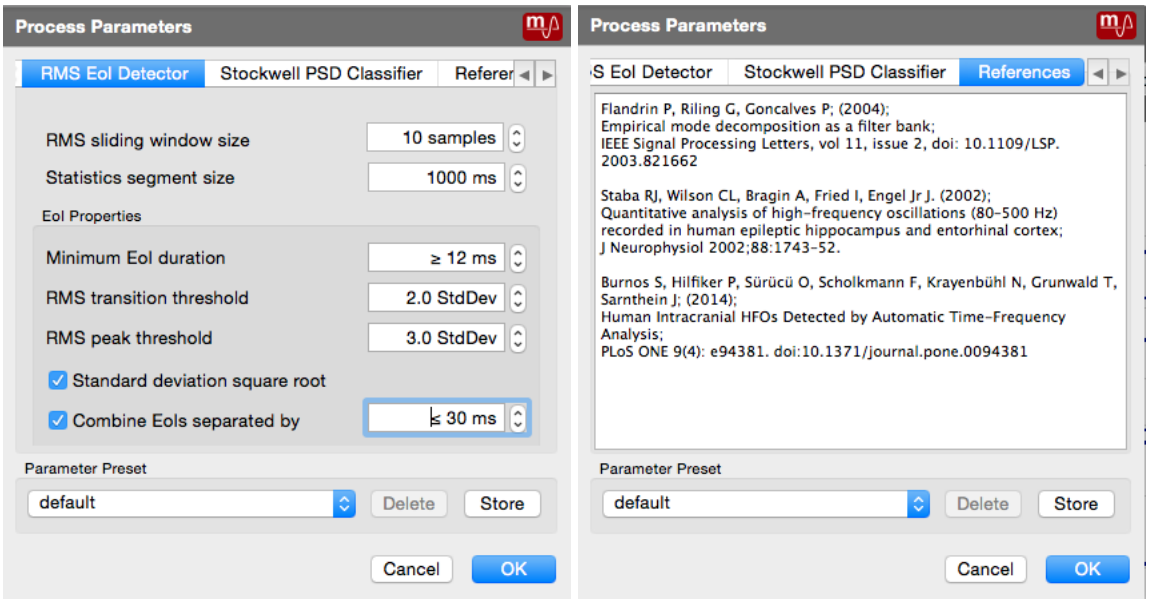

Figure S12. Process parameters adjustment dialog. RMS event detector parameter panel currently selected (left). References panel (right).

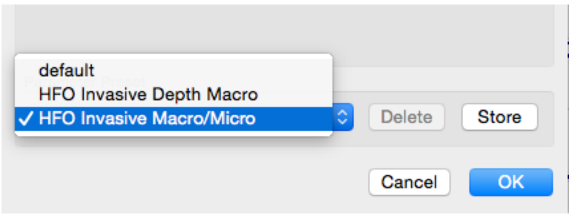

Figure S13. Store and recall of process parameter sets.

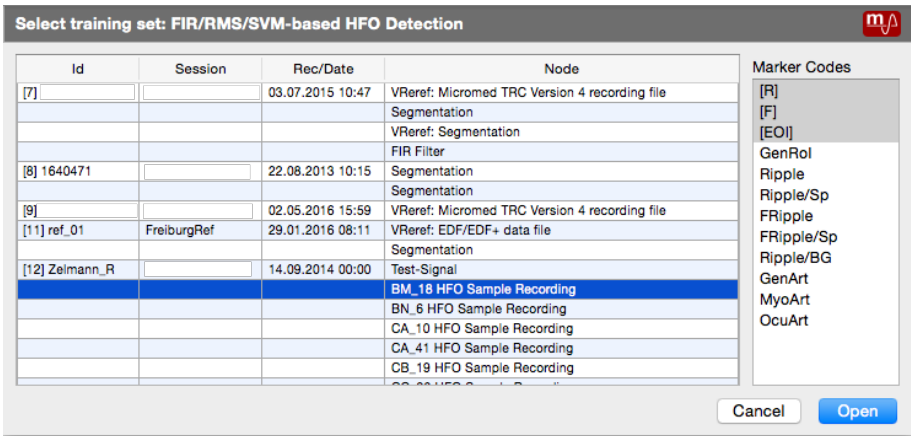

Figure S14. Dialog to select data nodes to be included (left listbox) and marker types to be considered (right listbox) in model generation or validation process. Patient IDs and session names partly removed.

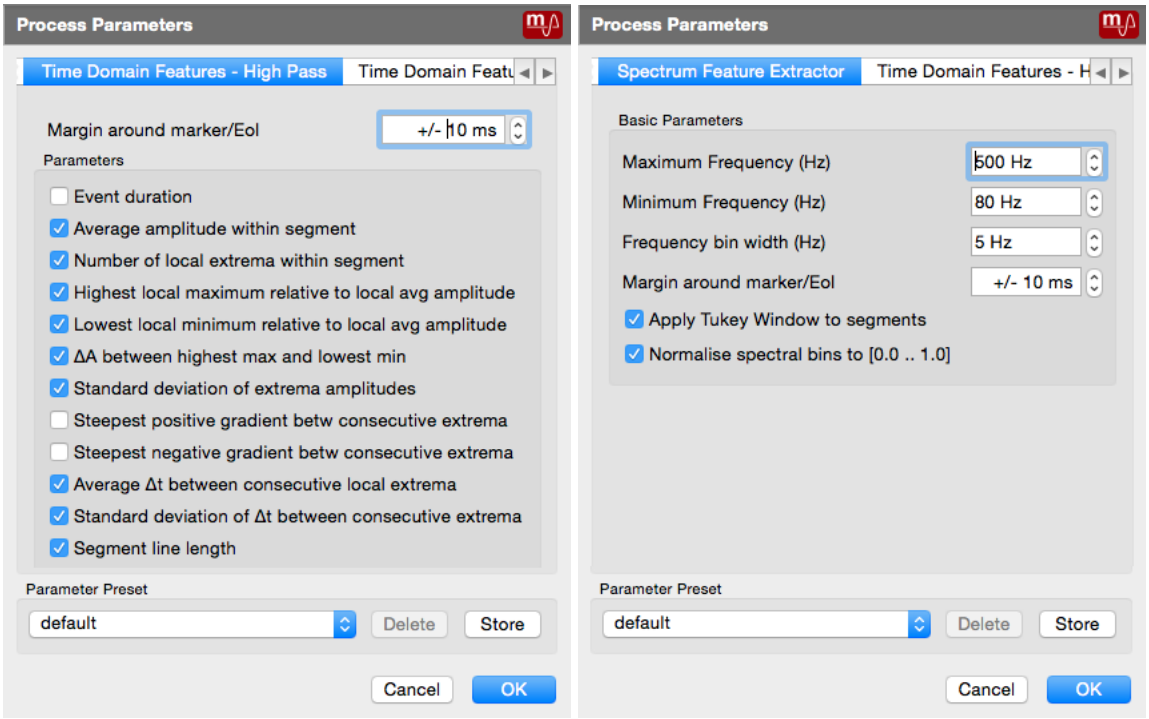

Figure S15. Parameter configuration panels for time domain related (left) and spectrum derived features (right).

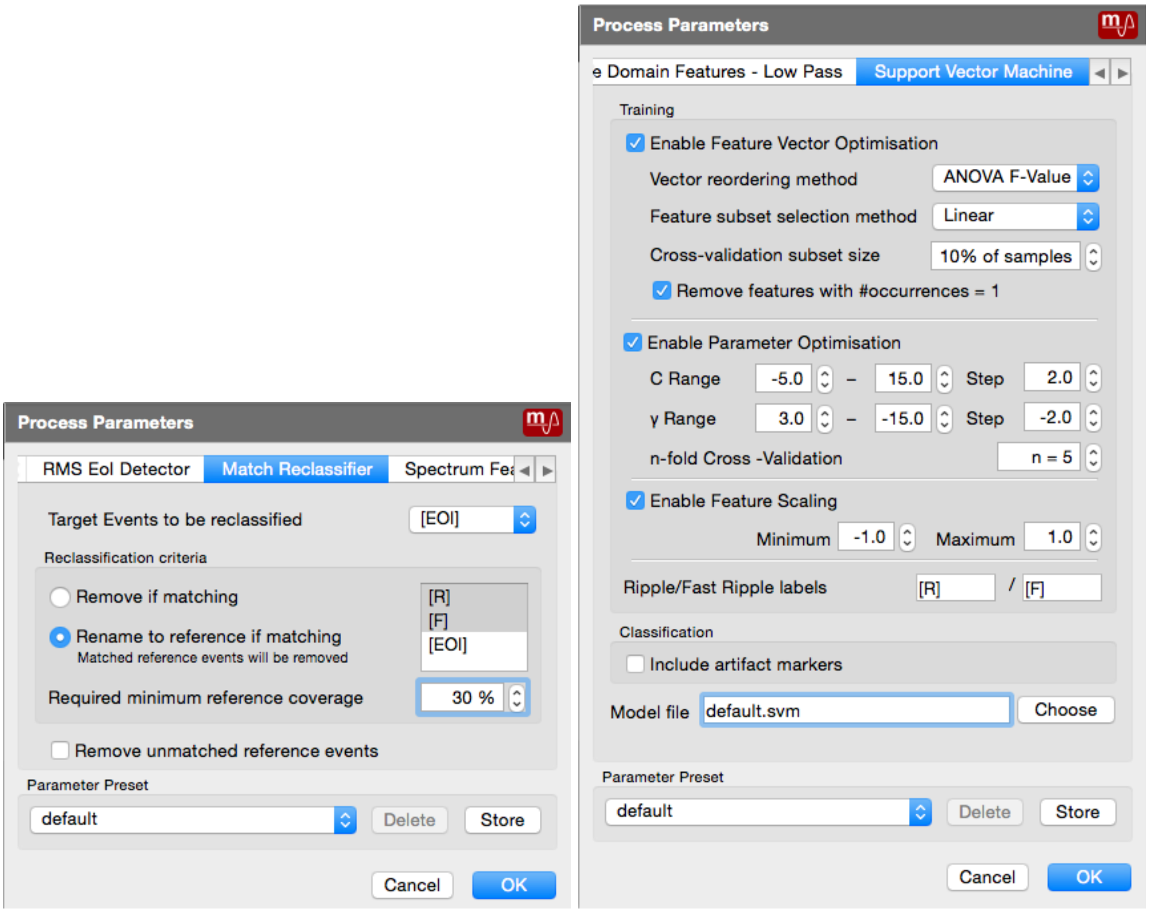

Figure S16. Configuration panel for relabelling detected events in accordance with matching pre-defined markers (left). Configuration panel for SVM classifier (right).

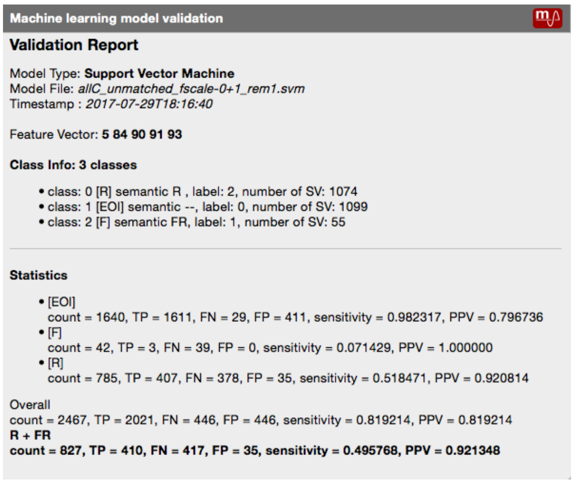

Figure S17. Exemplary machine learning model validation report.

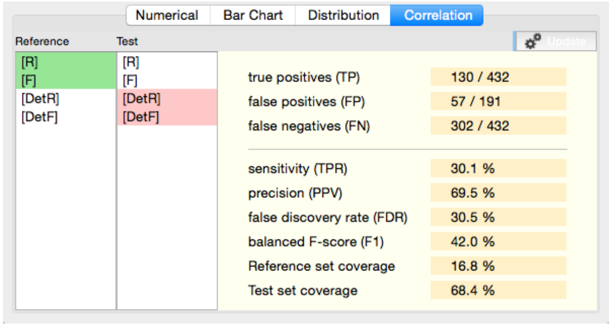

Figure S18. Example of statistical report on temporal and channel-wise correlation of selected types of events. Left column (green): reference subset; right column (red): test subset.

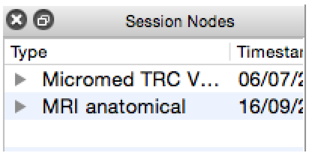

Figure S19. Imported MRI image set available in the data nodes dock window.

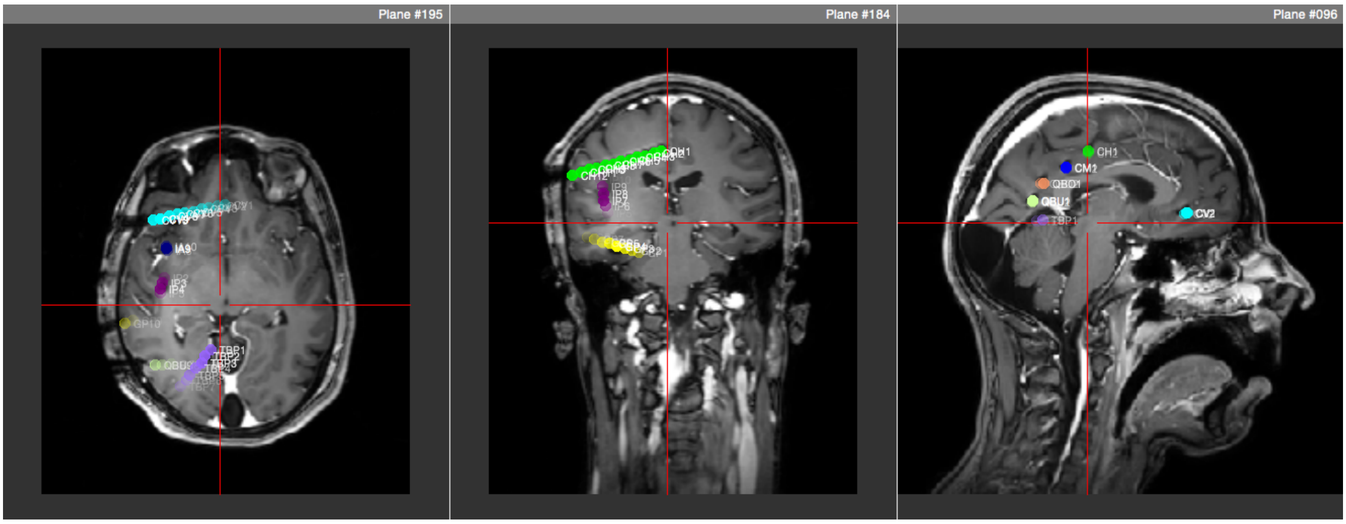

Figure S20. Planar visualisation of anatomical MRI in reduced scale. Left: transversal plane, middle: coronar/frontal plane, right: sagittal plane.

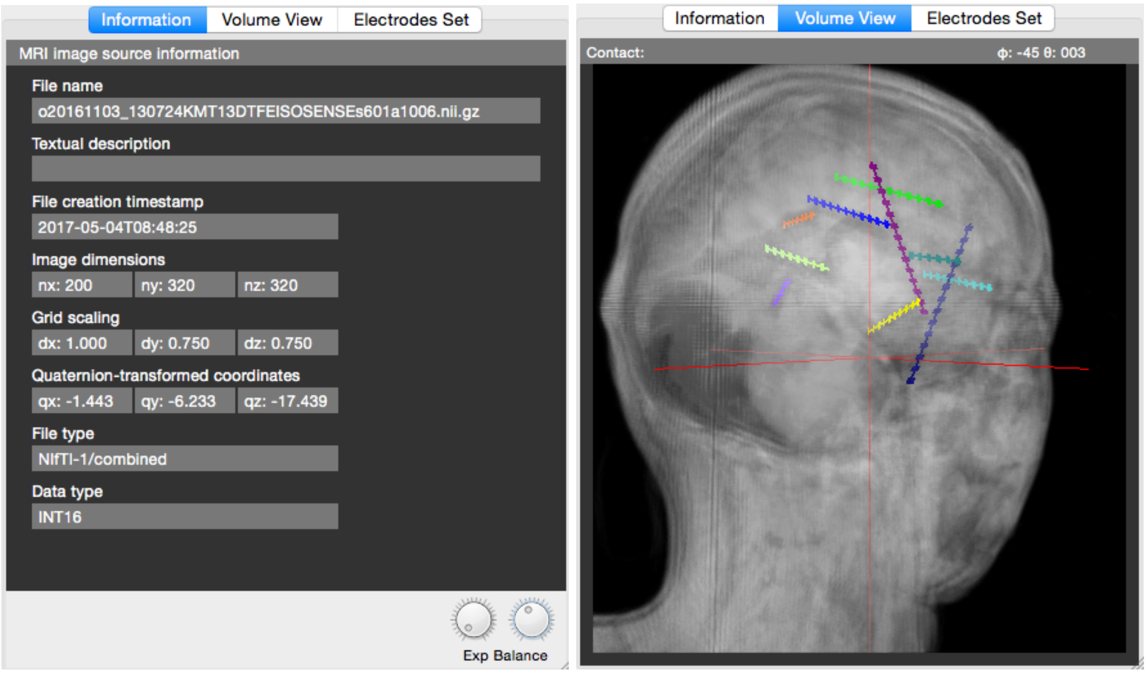

Figure S21. MRI image information panel (left). Three-dimensional MRI image projection (left).

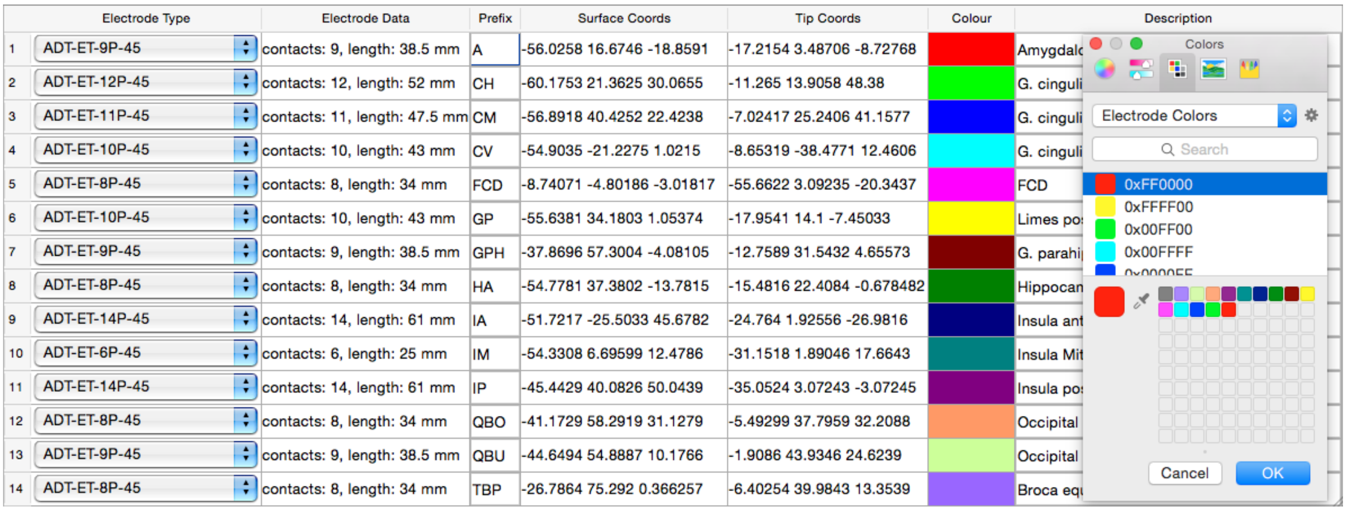

Figure S22. Electrodes set data node window showing a set of 14 electrodes. Right side: electrode colour selection dialog.

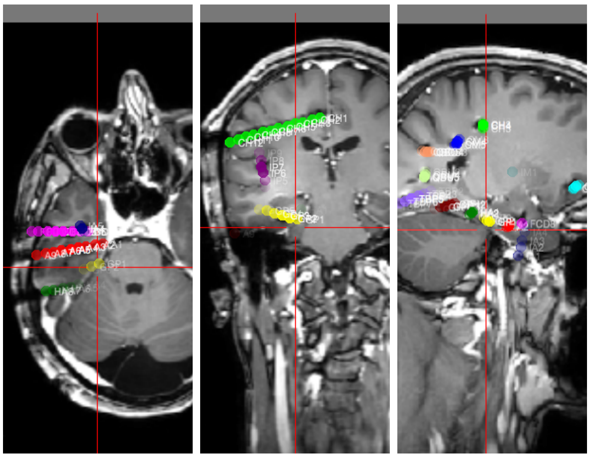

Information	Volume View	Electrodes Set				
	**Electrode Type**	**Prefix**	**Surface Coords**	**Tip Coords**	**Colour**	**Description**
1	ADT-ET-9P-45	A	-59.7:18.0:-20.7	-17.2 3.5 -8.7		Amygdalon
2	ADT-ET-12P-45	CH	-60.2 21.4 30.1	-11.3 13.9 48.4		G. cinguli post
3	ADT-ET-11P-45	CM	-56.9 40.4 22.4	-7.0 25.2 41.2		G. cinguli med
4	ADT-ET-10P-45	CV	-54.9 -21.2 1.0	-8.7 -38.5 12.5		G. cinguli ante
5	ADT-ET-8P-45	FCD	-8.7 -4.8 -3.0	-55.7 3.1 -20.3		FCD
6	ADT-ET-10P-45	GP	-55.6 34.2 1.1	-18.0 14.1 -7.5		Limes posteric
7	ADT-ET-9P-45	GPH	-37.9 57.3 -4.1	-12.8 31.5 4.7		G. parahippoc
8	ADT-ET-8P-45	HA	-54.8 37.4 -13.8	-15.5 22.4 -0.7		Hippocampus
9	ADT-ET-14P-45	IA	-51.7 -25.5 45.7	-24.8 1.9 -27.0		Insula anterior
10	ADT-ET-6P-45	IM	-54.3 6.7 12.5	-31.2 1.9 17.7		Insula Mitte
11	ADT-ET-14P-45	IP	-45.4 40.1 50.0	-35.1 3.1 -3.1		Insula posteric
12	ADT-ET-8P-45	QBO	-41.2 58.3 31.1	-5.5 37.8 32.2		Occipital supe
13	ADT-ET-9P-45	QBU	-44.6 54.9 10.2	-1.9 43.9 24.6		Occipital inferi
14	ADT-ET-8P-45	TBP	-26.8 75.3 0.4	-6.4 40.0 13.4		Broca equivale

Figure S23. Electrode coordinates definition using planar MRI views (clipped) and "Electrodes Sets" panel of information sub-window.
